# The Effect of Maternal Multiple Micronutrient Supplementation on Cognition and Mood during Pregnancy and Postpartum in Indonesia: A Randomized Trial

**DOI:** 10.1371/journal.pone.0032519

**Published:** 2012-03-12

**Authors:** Elizabeth L. Prado, Michael T. Ullman, Husni Muadz, Katherine J. Alcock, Anuraj H. Shankar, SUMMIT Study Group

**Affiliations:** 1 SUMMIT Institute of Development, Mataram, Nusa Tenggara Barat, Indonesia; 2 Psychology Department, Lancaster University, Lancaster, Lancashire, United Kingdom; 3 Program in International and Community Nutrition, University of California Davis, Davis, California, United States of America; 4 Neuroscience Department, Georgetown University, Washington, D.C., United States of America; 5 Center for Research in Language and Culture, Mataram University, Mataram, Nusa Tenggara Barat, Indonesia; 6 School of Public Health Department of Nutrition, Harvard University, Boston, Massachusetts, United States of America; The University of Adelaide, Australia

## Abstract

Maternal caregiving capacity, which is affected in part by cognition and mood, is crucial for the health of mothers and infants. Few interventions aim to improve maternal and infant health through improving such capacity. Multiple micronutrient (MMN) supplementation may improve maternal cognition and mood, since micronutrients are essential for brain function. We assessed mothers who participated in the Supplementation with Multiple Micronutrients Intervention Trial (SUMMIT), a double-blind cluster-randomized trial in Indonesia comparing MMN supplementation to iron and folic acid (IFA) during pregnancy and until three months postpartum. We adapted a set of well-studied tests of cognition, motor dexterity, and mood to the local context and administered them to a random sample of 640 SUMMIT participants after an average of 25 weeks (SD = 9) of supplementation. Analysis was by intention to treat. Controlling for maternal age, education, and socio-economic status, MMN resulted in a benefit of 0.12 *SD* on overall cognition, compared to IFA (*95%CI* 0.03–0.22, *p* = .010), and a benefit of 0.18 *SD* on reading efficiency (*95%CI* 0.02–0.35, *p* = .031). Both effects were found particularly in anemic (hemoglobin<110 g/L; overall cognition: *B* = 0.20, 0.00–0.41, *p* = .055; reading: *B* = 0.40, 0.02–0.77, *p* = .039) and undernourished (mid-upper arm circumference<23.5 cm; overall cognition: *B* = 0.33, 0.07–0.59, *p* = .020; reading: *B* = 0.65, 0.19–1.12, *p* = .007) mothers. The benefit of MMN on overall cognition was equivalent to the benefit of one year of education for all mothers, to two years of education for anemic mothers, and to three years of education for undernourished mothers. No effects were found on maternal motor dexterity or mood. This is the first study demonstrating an improvement in maternal cognition with MMN supplementation. This improvement may increase the quality of care mothers provide for their infants, potentially partly mediating effects of maternal MMN supplementation on infant health and survival. The study is registered as an International Standard Randomized Controlled Trial, number ISRCTN34151616. http://www.controlled-trials.com/ISRCTN34151616

## Introduction

Women's capacity to provide care for themselves and their families is crucial for their health and the health of their infants. Factors underlying the effectiveness of maternal caregiving include knowledge, such as signs of illness and actions necessary, perceiving and processing the observed situation, reasoning and decision-making, and monitoring the effectiveness of decisions in order to adapt to future situations. Although some interventions focus on promoting health knowledge, the possibility of improving infant health and survival by targeting these other skills essential for effective caregiving has received little attention. One way to improve these skills may be to improve the cognitive abilities that underlie and influence them, such as attention, memory, and mood.

Maternal multiple micronutrient (MMN) supplementation is one intervention that is likely to improve maternal cognition and mood, since micronutrients are essential for brain function (see below). Maternal MMN supplementation has been found to reduce the risk of low birthweight [1] and early infant mortality (<90 days after birth) [2] in developing countries, including Indonesia. The mechanisms of these effects are not yet fully understood. They may be partly due to factors such as improved placental development and improved transfer of nutrients to the fetus with MMN supplementation. These effects may also be partly due to neurological effects of MMN in pregnant and postpartum mothers, which may improve cognition and mood and therefore maternal caregiving capacity. For example, improved cognition and/or mood may promote maternal reasoning and decision-making, attentive and appropriate caregiving, and understanding of health information. Therefore, part of the effect of MMN supplementation on infant outcomes may be mediated by improvements in maternal cognition and mood, thereby increasing a mother's ability to care for herself and her infant.

The positive relationship between maternal education and infant and child health and survival [3], [4] highlights the importance of maternal capacity for children's health. Cognition improves with increasing years of education [5]. The increased cognitive capacity of more highly educated mothers may be the crucial factor that allows them to care more effectively for their children, thus promoting their health and survival. In support of this argument, higher maternal intelligence, independent of education, also reduces the risk of infant mortality [6] and infant malnutrition [7], as well as morbidity and poor physical growth in school age children [8], [9]. Higher maternal intelligence is also associated with positive caregiving behavior, such as more extensive and longer duration of breast feeding [10] and higher quality dietary intake in infants and toddlers, even after controlling for family socio-economic status and maternal education [11], [12], [13]. Maternal caregiving practices are also associated with maternal mood, for example, reduced preference for breastfeeding [14] and less sensitive mother-infant interaction [15] in mothers with depressive symptoms.

Although child health outcomes have been linked to measures of global maternal intelligence in the studies mentioned above, here, we focus on underlying cognitive abilities (e.g., declarative memory, attention and working memory, visuospatial ability; see below) for several reasons. First, intelligence depends in part on these specific functions, and assessing specific abilities allows analysis on both overall cognition and performance on specific tests. Second, we selected tests that have been linked to particular brain structures and mechanisms likely to be affected by micronutrient deficiency and other factors (see Method). For example, zinc plays an important role in brain areas (the hippocampus) and mechanisms (NMDA receptors) that subserve declarative memory [16], which underlies learning facts, events and words [17], [18]. Dopamine, serotonin, and noradrenaline synthesis all depend on iron, vitamin B6, vitamin B12, and folic acid [19], [20], [21]. Dopamine activity in the frontal lobes and basal ganglia plays a role in working memory (the temporary storage and manipulation of items), attention, executive function (the control and management of cognitive processes), and motor control [22], [23]. Noradrenaline is also important for attention, and both noradrenaline and serotonin are implicated in mood disorders [24]. Thus, deficiencies in these micronutrients may affect these particular cognitive functions.

With these potential mechanisms in mind, the present study was designed to examine the effects of the Supplementation with Multiple Micronutrients Intervention Trial (SUMMIT) on both overall maternal cognitive ability and specific cognitive functions. SUMMIT was a double-blind cluster-randomized trial carried out on the Indonesian island of Lombok by the University of Mataram, the Government of Nusa Tenggara Barat Province, the Ministry of Health of Indonesia, and Helen Keller International, comparing maternal MMN supplementation to iron and folic acid (IFA) [2]. We investigated the maternal cognitive outcomes of SUMMIT first for overall cognitive ability, calculated over the combined scores from the tests of the specific cognitive functions, and second for each individual test in the battery, which included tests of declarative memory, attention and working memory, mental rotation, category fluency, speeded picture naming, and reading efficiency, as well as motor dexterity and mood. Additionally, we examined the effects of MMN in two pre-specified subgroups of participants who had shown especially large reductions in early infant mortality from MMN supplementation [2]: those who were undernourished at enrollment (mid-upper arm circumference <23.5 cm) and those who were anemic at enrollment (hemoglobin concentration <110 g/L).

## Methods

### SUMMIT Design

The design and procedures of SUMMIT are reported in detail elsewhere [2], and are only summarized here. The protocol for this trial and supporting CONSORT Checklist are available as supporting information; see Checklist S1 and Protocol S1. Pregnant women throughout the Indonesian island of Lombok were enrolled in SUMMIT from July, 2001 to April, 2004. Enrollment took place at local pre-natal care clinics run by midwives trained and employed by the Indonesian Ministry of Health. SUMMIT staff attended these clinics and reviewed the informed consent form with women upon confirmation of pregnancy. Written informed consent was obtained from all participants. Consenting women received a daily supplement throughout the duration of pregnancy and until three months post-partum. Midwives were randomly assigned to distribute either MMN or IFA; thus, all women who received pre-natal care from the same midwife received the same supplement. The contents of both supplements are reported in supporting information Appendix S1. All supplements were coded at the manufacturing plant; hence all SUMMIT scientists and personnel, government staff, and participants were unaware of the allocation of MMN and IFA. Ethical approval for the study protocol and informed consent form was obtained from the National Institute of Health Research and Development of the Ministry of Health of Indonesia, the Provincial Planning Department of Nusa Tenggara Barat Province, and the Johns Hopkins Joint Committee on Clinical Investigation, Baltimore, USA. The study is registered as an International Standard Randomized Controlled Trial, number ISRCTN34151616 (http://www.controlled-trials.com/ISRCTN34151616).

Blood samples were drawn both before and after supplementation in a random subsample of 2,369 SUMMIT participants. This subsample was divided into four groups, each of which provided a blood sample at enrollment in SUMMIT and at one of four subsequent time points: one month after enrollment, 36 weeks of gestation, one week post-partum, and 12 weeks post-partum. Participants in the subsample were targeted to participate in cognitive testing. Note that blood analyses are not discussed in the present paper.

### Participants in Cognitive Research

**Figure 1:**
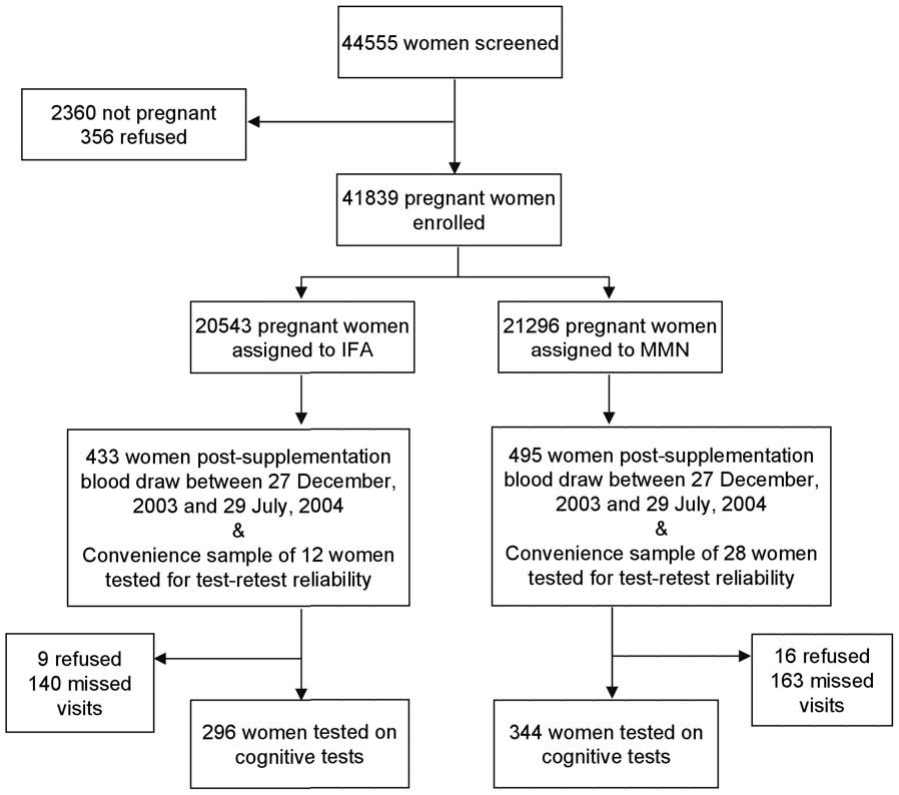
Trial Profile.

Figure 1 shows the trial profile. All participants who had their second blood draw between 27 December, 2003 and 29 July, 2004 were targeted for cognitive testing as close as possible to the time of their second blood draw. An additional convenience sample of 40 SUMMIT participants was tested to establish the test-retest reliability of the final version of each test, after the completion of pilot testing. The results of reliability testing indicated that no further revisions were required, therefore, data from all 640 participants were included in the analyses reported here. The results of analyses with and without the 40 participants assessed for reliability testing were similar.

Of the total participants targeted (*n* = 968), the proportion that was not tested (because the woman refused or the visit was missed for logistical reasons) was not different between IFA (149/445) and MMN (179/523; *p* = .808). Moreover, the 640 participants did not significantly differ from the 328 who were not tested in any of the baseline characteristics (see Results), suggesting that these were missing at random.

The average amount of time between enrollment (when participants began taking the supplements) and cognitive testing was 25.2 weeks (*SD* = 9.1). We tested 49% of the participants during pregnancy (*mean* = 71.8 days before birth, *SD* = 51.2) and 51% in the 6 months after birth (*mean* = 102.5 days after birth, *SD* = 46.5).

### Test Selection

Our selection of cognitive, motor, and mood tests was based on a number of factors. We selected tests that (1) are designed to primarily tap aspects of specific cognitive or other brain-related functions, particularly those that are important for daily life, that may be affected by micronutrient deficiency (see Introduction), and that may change in pregnancy (see Discussion); (2) have been tied to particular brain structures and mechanisms; (3) are well-established and widely used; (4) do not require special equipment (only paper, pencil, stopwatch, and tape recorder); (5) do not require literacy (except for the reading efficiency test); and (6) are easily administered and scored, and do not require subjective judgments from the testers.

Table 1 shows the tests used in this study. For each test the table presents a summary of the methods as well as brief descriptions of the main cognitive and other function(s) the test probes, and the main brain areas that the test depends on.

**Table 1:** Cognitive, Motor, and Mood Tests.

Test	Method and Scores	Main Underlying Cognitive and Brain Systems
Word List Memory Test	Participants were asked to immediately recall a target list of eleven orally presented words three times, followed by an interference trial requiring the immediate recall of a second eleven-word list, and then a request to recall the first list (*initial recall score*: number of items recalled). Then, after an average delay of 18 minutes (during which other tests were given), participants were again asked to recall the initial list (*delayed recall score*: number recalled), and then given a recognition test (*delayed recognition score: z*-score for the proportion of words correctly identified as on the target list minus the *z*-score for the proportion of words incorrectly identified as on the list).	This test was based on the Rey Auditory Verbal Learning Test (RAVLT) [66], which is a widely-used test of declarative memory (learning) ability. The declarative memory system depends mainly on the hippocampus and related medial temporal lobe structures [17].
Digit Span Forward and Backward Tests	In both of these tests, participants were orally presented with increasingly longer sequences of digits, and had to either repeat them (digit span forward) or repeat them backwards (digit span backward), until an error was committed on two consecutive trials of the same length. For each test, the score was the total number of sequences repeated without errors.	The digit span forward and backward tests were based on the digit span subtests of the Wechsler Adult Intelligence Scale III [67]. The digit span forward test measures attention, freedom from distractibility, and verbal short-term retention capacity [68]. Performance on this test involves the inferior supramarginal gyrus and cerebellum, among other areas [69], [70], [71]. Digit span backward involves manipulation as well as retention of items in working memory, which is rooted in areas of the prefrontal and parietal cortex [72].
Mental Rotation Test	The participant was visually presented with five rows of figures. For each row, participants were instructed to mark the figures that were rotations but not mirror images of the target figure. The score was calculated as the *z*-score for the proportion of figures correctly identified as rotations minus the *z*-score for the proportion of mirror images incorrectly marked as rotations.	This test was based on the Card Rotations Test from the Kit of Factor-Referenced Tests produced by the Educational Testing Service [73]. Mental rotation assesses visuospatial ability and dynamic mental imagery, and activates areas in the parietal lobe and other structures [74], [75].
Category Fluency Test	The score on this test was the total number of appropriate words participants were able to produce in a given category in one minute, in any Sasak dialect or in Indonesian. Two trials were administered: the category *food* and the category *people's names*.	Category fluency taps semantic memory, which is rooted in areas of the temporal lobe [68]. It also involves executive function, rooted in areas of the frontal lobe [76].
Speeded Picture Naming Test	Participants were instructed to point to and say out loud the name of each picture on a page, in order (from left to right, top to bottom), as quickly and accurately as possible. The score was calculated as the time to complete the page divided by the number of pictures correctly named.	Picture naming depends on lexical (word) abilities and semantic memory. Speeded picture naming predicts reading performance in young children, and activates brain areas implicated in reading, including the inferior frontal cortex, temporo-parietal areas, and the ventral visual stream [77].
Reading Efficiency Test	Participants were instructed to read aloud a list of real words and then a list of phonologically plausible pseudowords, as quickly and accurately as possible as they pointed to each one. The score for each of the two lists was calculated as the time to complete the list divided by the number of (pseudo)words correctly read aloud.	This test was based on the Test of Word Reading Efficiency [78]. Reading (pseudo)words aloud is a complex function that draws on a number of structures throughout the brain [79], [80].
Coin Rotation Test	Participants were instructed to rotate a plastic coin as fast as possible in 10 seconds, using the thumb, index and middle fingers, first in the right hand and then in the left. The score for each hand was the number of times the participant rotated the coin in the 10 second period.	This test assesses motor dexterity. Performance depends on intact sensory-motor function and on the brain areas underlying motor control, which include portions of the basal ganglia and frontal cortex, especially the primary motor cortex, as well as the cerebellum [81], [82]. Motor function also depends on dopamine activity in the basal ganglia [23].
Mood Scale	The items in this test probe feelings of depressed mood, guilt and loneliness, hopelessness, loss of appetite, and sleep disturbance. In an interview format, participants rated the frequency of these symptoms over the past week on a scale of 0-3. The score was calculated as the sum of the item ratings after reversing negative items, such that a higher score indicated more positive mood.	This test was based on the Center for Epidemiological Studies Depression Scale [83]. Depression is related to the function of the neurotransmitters serotonin, dopamine, and noradrenaline [84]. Mood is regulated by various brain structures, including in the limbic system [85].

### Test Adaptation and Evaluation

The tests were adapted to the local language, culture and setting in Lombok following the principles presented in Prado, Hartini, Rahmawati, et al. (2010) [25]. We describe the adaptation and evaluation of the maternal cognitive tests in the following paragraphs.

#### Test translation and stimulus development

Test sessions were conducted in Sasak, which is the predominant spoken language in Lombok, although it is rarely written. Test instructions were translated from English to Indonesian, and printed on the testing forms in Indonesian, the medium of academic (and literacy) instruction in Lombok; this allowed the testers to read the instructions comfortably and translate them slightly differently into various dialects of Sasak as they were testing. Test stimuli, consisting of words and pictures, were developed through various surveys. For example, a dialect survey was conducted to confirm that the Sasak word stimuli in the Word List Memory Test and Speeded Picture Naming Test were the same across all dialects of Sasak. In addition, subjective frequency and imageability (how easily a mental picture can be generated) ratings were gathered to arrange and balance items within tests, for example to ensure that the target list of the Word List Memory Test included some high frequency, highly imageable words that would be easy to remember (e.g. *oil*) and some lower frequency and imageability words that would be more difficult to remember (e.g. *opponent*).

#### Reliability testing

A series of pilot tests was conducted in order to adapt and revise the instructions, items and procedures of the tests for use in Lombok, as well as to determine whether or not each test was suitable for the local context. Some tests, which are not discussed here, were piloted and discarded because of low test-retest reliability or lack of feasibility under the testing conditions in rural villages.

The final version of each test was evaluated for three types of reliability: test-retest reliability, internal consistency, and inter-tester reliability. To evaluate test-retest reliability, three certified testers (see below), each administering one of the three sets of tests (see below), were assigned to visit a group of participants (ranging from 9 to 18) twice (the average time between visits was 4 days, ranging from 2–16 days). The test-retest reliabilities ranged from *r* = .44 to .96. Internal consistency was calculated on the full sample of participants tested (*N* ranging from 300 to 501 for the various tests) and ranged from *Cronbach's Alpha* = .67 to .87. To evaluate inter-tester reliability, all seven testers were periodically assigned to revisit a participant who had previously been tested by a different tester, in order to ensure that the data was being collected consistently across testers. Inter-tester reliability ranged from *r* = .49 to *r* = .97.

### Procedure

#### Testers

Seven testers, all of whom were native speakers of Sasak and fluent in Indonesian, were recruited and trained to administer the tests. After training, they were required to pass a written exam and a field evaluation before being certified to administer the tests.

#### Sets of tests

The large number of tests in the final battery precluded administering all tests in one visit. In addition to the tests listed in Table 1, tests of near vision, distance vision, and audition were also administered, further lengthening the test session. Most participants scored at ceiling on these tests, precluding meaningful analysis, therefore the data from these tests are not reported here. To reduce the length of the test session, we divided the tests into three sets: X (Word List Memory and Digit Span Forward and Backward), Y (Category Fluency, Coin Rotation, and Mood Scale) and Z (Speeded Picture Naming, Reading Efficiency, and Mental Rotation), and administered two sets to each participant. Two testers administered sets X and Y to 136 participants (21%), three testers administered sets X and Z to 333 (52%), and two testers administered sets Y and Z to 171 (27%). Different numbers of testers administering each test led to unequal numbers of participants in each group.

#### Quality control

All test sessions, which were conducted at participants' homes, were audio-recorded, and the tapes and forms reviewed in three steps. First, the tape was reviewed by the tester who administered the tests, in order to complete and check the form. Second, the tape was reviewed by a different tester who had not been present at testing, who corrected any further discrepancies between the tape and the form. Third, 10% of the tapes and corresponding forms were reviewed by a supervisor (the first author) to ensure the quality of the testers' work, which was in fact consistently high. The data was then entered into SPSS. Every form was double-entered and any discrepancies were corrected.

### Statistical Analyses

#### Group characteristic comparisons

Participants who received MMN were compared to those receiving IFA on the following baseline characteristics (i.e., at enrollment): age (years), education (completed years of formal education), and three indicators of nutritional status (height in cm, mid-upper arm circumference <23.5 cm, and hemoglobin concentration <110 g/L, based on capillary blood from finger prick). The two groups were also compared on an index of socio-economic status, derived from a survey administered at baseline concerning whether or not the family owned certain household items. Out of a total of twelve items, four items were discarded that did not correlate with the total (owned a horsecart, a boat, a house, and a vendor's pushcart; *r*s<.1). Two items were also eliminated due to low variability (only six participants owned a car and four owned a satellite dish). Removing these six items improved *Cronbach's Alpha* from 0.49 to 0.59, The six remaining items (owned a radio, a TV, a refrigerator, a bike, a motorbike, and a small sales business) were summed for a total score. This score was coded as a multinomial variable with four levels: 0, 1, 2 and >2.

In addition, the two groups were compared on the amount of time between enrollment and cognitive testing (in weeks); mean compliance in taking the supplement (percentage of supplements consumed); when the participant was tested (first, second, or third trimester, or after birth); hours of sleep in the 24 hours before testing; and the set of tests given to the participant (XY, XZ, or YZ). Comparisons between the two groups were made for all participants for whom this information was available.

For the continuous variables, the difference between participants who received IFA and MMN was estimated in mixed effects models using SAS PROC MIXED. Mixed effects models are similar to multiple regression models, but allow for the specification of both fixed and random effects. The model was specified with a fixed effect of supplement type and a random effect of midwife code on intercept. This random effect was included in all analyses since the randomization of IFA and MMN was allocated by midwife rather than by individual participant; specification of midwife code as a random effect accounts for any variation between midwife clusters [26]. All statistical analyses using SAS PROC MIXED (here and below) were estimated using an unstructured covariance structure, and degrees of freedom were estimated using the Satterthwaite method [27]. For the multinomial variables, the difference was estimated in generalized linear models using SAS PROC GENMOD with midwife code as a repeated measure, with a multinomial distribution, and with the cumlogit link function. For the binomial variables, the difference was estimated using SAS PROC GENMOD with midwife code as a repeated measure, and with a binomial distribution and the log link function.

#### Overall cognition

The analysis of the effects of MMN on overall cognition included all cognitive test scores (i.e., without the tests of motor ability and mood): Word List Memory, Digit Span Forward and Backward, Mental Rotation, Category Fluency, Speeded Picture Naming, and Reading Efficiency. The timed tests (Speeded Picture Naming and Reading Efficiency) resulted in skewed distributions. These scores were reciprocal transformed, reducing skewness (to <1) and changing the direction of the scores to match the other scores (higher is better). For all other tests, skewness <1. For each test score, *z*-scores were computed based on the distribution of all participants in this study. Rather than averaging the *z*-scores for each participant, all test *z*-scores from every participant were entered as the dependent variable in a nested mixed effects model with random effects of both midwife code and participant on intercept. Specification of a random effect of participant is an alternative to averaging the *z*-scores for each participant that accounts for variation between participants without the loss of information that occurs when averaging [28].

This model was estimated (and is reported below) first with a fixed effect of *supplement type* as the only independent variable, and second with fixed effects of *supplement type* and four covariates: *set of tests*, education, age, and socio-economic index. The set of tests given to the participants was included as a covariate in order to account for any variation between participants due to taking different (but overlapping; see above) sets of tests. The other three covariates were chosen based on their relationship with the full set of cognitive test *z*-scores. Each of the group characteristic variables in Tables 2 and 3 was considered for inclusion as a covariate by entering it individually (i.e., as the only fixed effect) into the same type of nested mixed effects model described just above (i.e., with random effects of midwife code and participant on intercept). Only the variables education, age, and socio-economic index predicted the cognitive *z*-scores (*p*s<.06; all other *p*s>.22) and were therefore included in secondary analyses.

**Table 2:** Group Characteristic Comparisons of Maternal Cognitive Participants Who Received IFA and MMN (Continuous Variables).

	IFA		MMN		IFA vs MMN	
Characteristic	*n*	*Mean (SD)*	*n*	*Mean (SD)*	*t*	*p*
Baseline age (years)	233	25.0 (5.5)	284	25.5 (5.8)	1.03	0.303
Baseline education (completed years of formal education)	236	6.7 (3.4)	288	6.7 (3.5)	0.33	0.741
Baseline height (cm)	92	149.6 (5.3)	96	149.6 (4.2)	0.13	0.898
Time between enrollment and cognitive testing (weeks)	295	25.3 (9.4)	343	25.1 (8.9)	0.12	0.905
Mean compliance (percentage of supplements consumed)	182	75.8 (25.6)	190	73.0 (25.7)	1.48	0.142
Hours of sleep in the 24 hours before testing	296	7.8 (1.9)	344	7.9 (1.9)	0.68	0.497

**Table 3:** Group Characteristic Comparisons of Maternal Cognitive Participants Who Received IFA and MMN (Categorical Variables).

		IFA		MMN		IFA vs MMN	
Characteristic		*n*	*Percent*	*n*	*Percent*	*z*	*p*
Baseline mid upper arm circumference <23.5 cm		33/93	35%	34/106	32%	0.51	0.611
Baseline haemoglobin concentration <110 g/L		50/106	47%	65/116	56%	1.27	0.203
Baseline socio-economic index						0.41	0.683
	0	75/236	32%	97/290	33%		
	1	71/236	30%	88/290	30%		
	2	48/236	20%	55/290	19%		
	>2	42/236	18%	50/290	17%		
When tested on cognitive tests						1.54	0.123
	1st trimester	4/222	2%	10/259	4%		
	2nd trimester	18/222	8%	13/259	5%		
	3rd trimester	76/222	34%	109/259	42%		
	After birth	124/222	56%	127/259	49%		
Set of tests given to the participant						0.09	0.925
	XY	63/296	21%	73/344	21%		
	XZ	153/296	52%	180/344	52%		
	YZ	80/296	27%	91/344	26%		

To examine effects in participants who were undernourished at enrollment (mid-upper arm circumference <23.5 cm) and participants who were anemic at enrollment (hemoglobin concentration <110 g/L), the interaction between each of these two variables and supplement type was (separately) added to the models (with and without the four covariates), and the effect of MMN was estimated for each subgroup [2].

#### Performance on individual tests

Composite scores for each cognitive test, as well as motor ability and mood, were calculated as the average of all *z*-scores from that test. For example, the Word List Memory score was calculated as the average of the *z*-scores for the initial recall score, the delayed recall score, and the delayed recognition score (see Table 1). For tests comprised of a single score (e.g., Speeded Picture Naming, Mental Rotation), the score was simply the *z*-score for that test.

The effect of MMN on each (composite or individual) test score was estimated and adjusted for clustered randomization using SAS PROC MIXED by specifying a fixed effect of supplement type and a random effect of midwife code. For three test scores (Word List Memory score, Speeded Picture Naming score, and Mood score), the estimate of the random effect of midwife code was zero; in these cases, PROC MIXED automatically estimated the model without this random effect.

## Results

### Group Characteristic Comparisons

None of the group characteristics described above differed significantly between participants who received IFA and MMN (Tables 2 and 3). The 640 participants did not differ from the 328 who were not tested (see Methods) in any of the 6 baseline characteristics in Tables 2 and 3 (*p*s>.05), suggesting that these were missing at random.

### Overall Cognition

**Figure 2:**
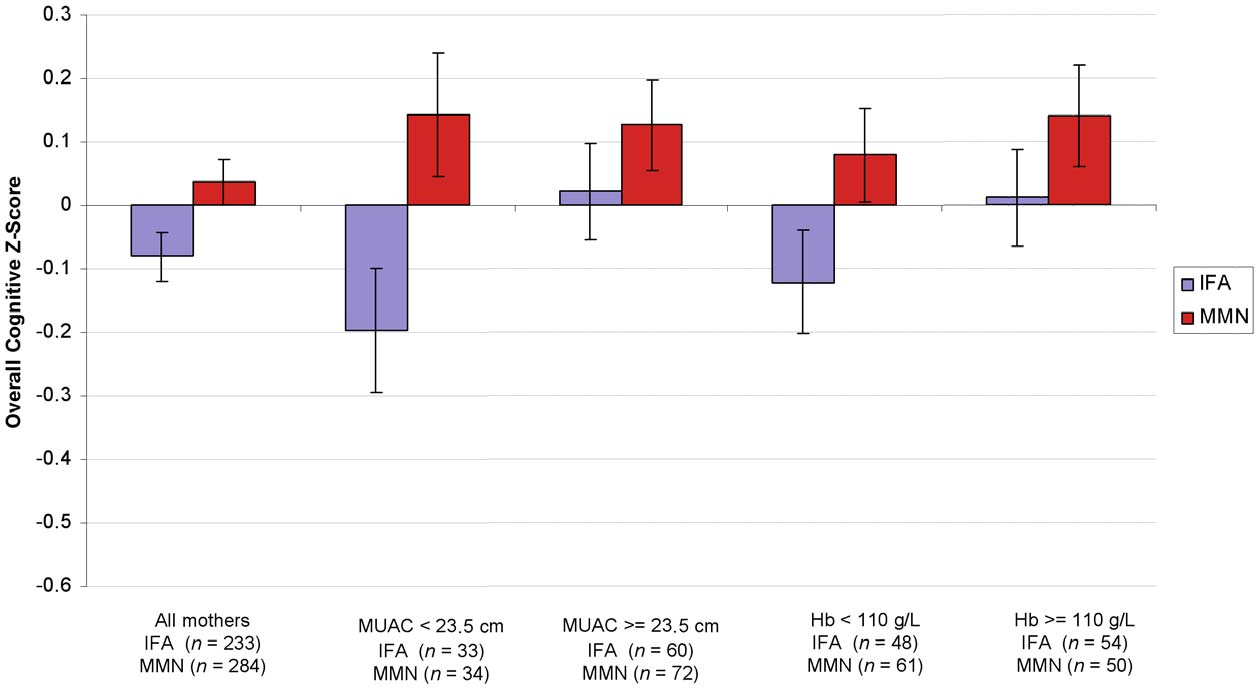
Mean cognitive *z*-score for mothers who received IFA and MMN overall and for each subgroup. Mean *z*-scores are adjusted for cluster randomization, education, age, socio-economic index, and set of tests. Error bars show the standard error of the mean.

The effect of MMN on overall cognitive *z*-scores in all participants and in each subgroup of participants is reported in Table 4 and depicted in Figure 2. The estimate (*B*) represents the unstandardized estimate of the difference between participants who received IFA and MMN, expressed as a fraction of the variation (standard deviation) of the cognitive score. For example, in the model including all 640 participants and no covariates, participants who received MMN scored an average of 0.12 standard deviations higher on the overall cognitive score (i.e., across all cognitive tests; see Methods) than participants who received IFA (*p* = .029). The model with the four covariates (education, age, socio-economic index and *set of tests*), which included the 517 participants for whom no values were missing for these covariates, resulted in a similar estimate of the effect of MMN (*B* = 0.12, *p* = .014; Table 4).

**Table 4:** The Effect of MMN on Overall Cognitive Z-Scores.

	Adjusted for Cluster Randomization			Adjusted for Cluster Randomization, set of tests, and maternal education, age, and socio-economic index		
	*n*	Estimate (95% CI)	*p*	*n*	Estimate (95% CI)	*p*
All participants	640	0.12 (0.01–0.23)	0.029	517	0.12 (0.03–0.22)	0.014
Mid-upper arm curcumference <23.5 cm	67	0.41 (0.09–0.72)	0.012	67	0.33 (0.07–0.59)	0.020
Mid-upper arm circumference > = 23.5 cm	132	0.04 (−0.18–0.27)	0.704	132	0.11 (−0.08–0.31)	0.248
Hemoglobin <110 g/L	115	0.23 (−0.01–0.47)	0.057	109	0.20 (0.00–0.41)	0.055
Hemoglobin > = 110 g/L	107	0.01 (−0.15–0.17)	0.775	104	0.13 (−0.08–0.33)	0.225

*Note*. The estimate of the effect of MMN represents the estimate of the difference in cognitive scores between participants who received IFA and MMN, expressed as a fraction of the variation (standard deviation) of the cognitive score. A positive estimate indicates that participants who received MMN scored higher than those who received IFA.

Among the 199 women for whom mid-upper arm circumference at enrollment was available, only those who were undernourished (mid-upper arm circumference <23.5 cm) showed a significant benefit of MMN on the cognitive scores (Table 4). Among the 222 women for whom hemoglobin concentration was available, only those who were anemic (hemoglobin <110 g/L) showed a benefit of MMN that approached significance (Table 4). Although mid-upper arm circumference and hemoglobin concentration were not collected from all participants, the proportion missing did not differ between IFA and MMN (*p*s>.57). In addition, cognitive scores did not differ between women for whom these data were missing and available (*p*s>.25), therefore it is unlikely that excluding those participants resulted in bias.

All of the covariates except the socio-economic index also significantly predicted the cognitive scores (*education*: *B* = 0.11, *t*(506) = 14.68, *p*<.001; *age*: *B* = −0.02, *t*(510) = 3.62, *p*<.001; *socio-economic index*: *F*(3,502) = 0.40, p = .750; *set of tests*: *F*(2, 276) = 4.71, *p* = .010). Note that these numbers are slightly different than those reported in the Analysis section. Here we report the results of the model in which supplement type and the four covariates were included as fixed effects together in the same model, while in the Analysis section, we report the results of the models in which each variable was entered individually as the only fixed effect in the model (i.e., with a separate model for each independent variable).

The estimate of the effect of education on cognition can be used as a reference for the magnitude of the effect of MMN. The linear estimate of the effect of education (*B* = 0.11) demonstrates that the cognitive *z*-scores increased by about 0.11 standard deviations with every additional year of formal education. This is similar to the estimate of the effect of MMN on the cognitive *z*-scores over all participants (0.12; Table 4), demonstrating that MMN supplementation improved cognition approximately the equivalent of one year of education.

These results confirm that maternal MMN supplementation improved overall cognitive function. Moreover, women with poorer nutritional status, as evidenced by low mid-upper arm circumference or anemia, benefited particularly from MMN supplementation.

### Performance on Individual Tests

**Figure 3:**
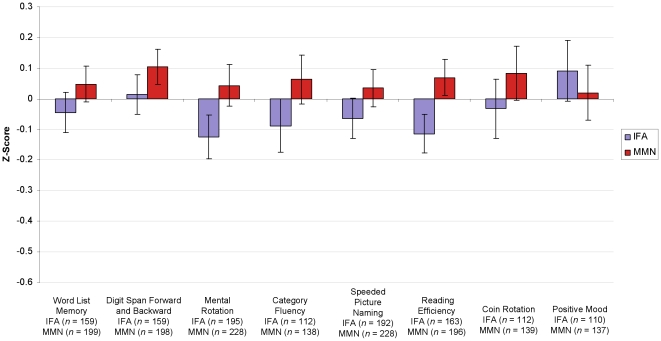
Mean z-score on each individual test for mothers who received IFA and MMN. Mean *z*-scores are adjusted for cluster randomization, education, age, socio-economic index, and set of tests. Error bars show the standard error of the mean.

The effect of MMN on the (composite or individual) score for each test is reported in Table 5 and depicted in Figure 3. Participants who received MMN scored higher than those who received IFA in every cognitive and motor test, although the effect of MMN only reached significance for the Reading Efficiency test (*p* = .031).

**Table 5:** The Effect of MMN on Each Composite or Individual Test Z-Score.

	Adjusted for Cluster Randomization			Adjusted for Cluster Randomization, set of tests, and maternal education, age, and socio-economic index		
	*n*	Estimate (95% CI)	*p*	*n*	Estimate (95% CI)	*p*
Word List Memory	469	0.12 (−0.04–0.29)	0.144	358	0.09 (−0.07–0.25)	0.253
Digit Span Forward and Backward	468	0.08 (−0.09–0.24)	0.366	357	0.09 (−0.07–0.25)	0.259
Mental Rotation	502	0.17 (−0.02–0.35)	0.083	423	0.17 (−0.02–0.36)	0.081
Category Fluency	306	0.14 (−0.06–0.34)	0.175	250	0.15 (−0.07–0.38)	0.186
Speeded Picture Naming	499	0.14 (−0.04–0.33)	0.124	420	0.10 (−0.07–0.27)	0.255
Reading Efficiency	426	0.20 (0.01–0.39)	0.040	359	0.18 (0.02–0.35)	0.031
Coin Rotation	307	0.05 (−0.19–0.28)	0.697	251	0.12 (−0.13–0.36)	0.364
Positive Mood	300	−0.09 (−0.32–0.14)	0.469	247	−0.07 (−0.32–0.18)	0.579

*Note*. The estimate of the effect of MMN represents the estimate of the difference in scores between participants who received IFA and MMN, expressed as a fraction of the variation (standard deviation) of the cognitive score. A positive estimate indicates that participants who received MMN scored higher than those who received IFA.

For the Mood Scale, participants receiving MMN showed slightly (non-significantly) lower scores, indicating somewhat depressed mood. This demonstrates that MMN supplementation did not improve mood during pregnancy and postpartum and suggests that enhanced mood cannot account for the cognitive improvements associated with MMN.

**Figure 4:**
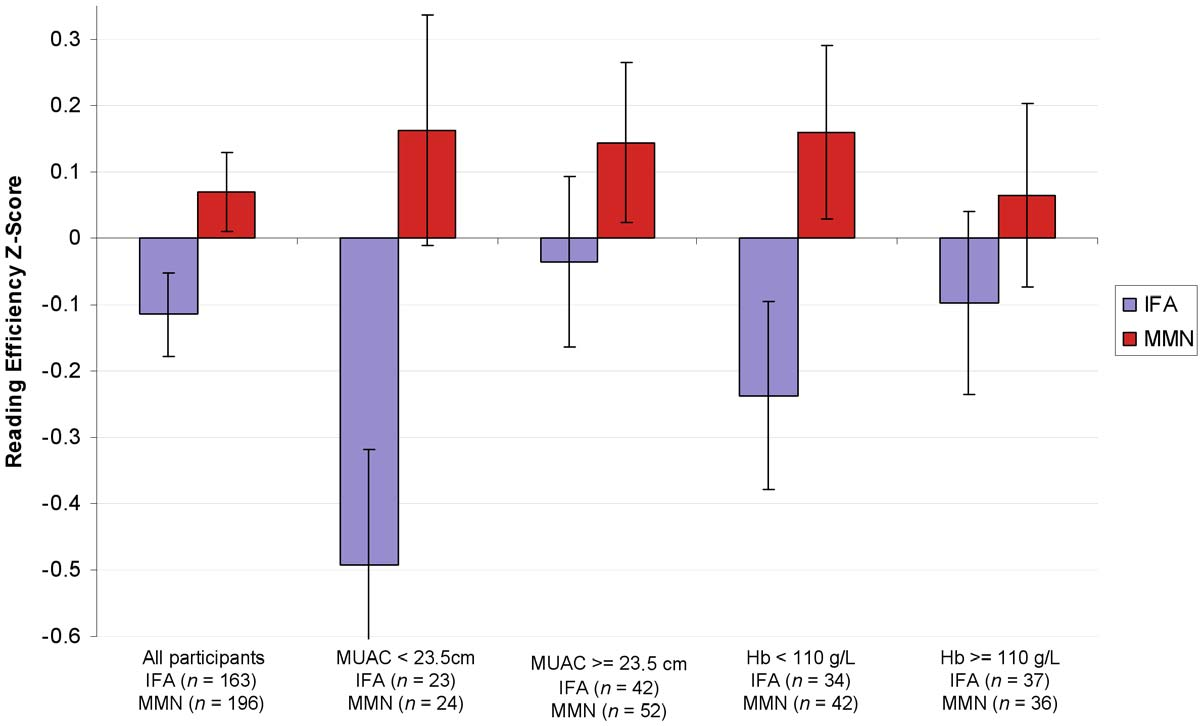
Mean Reading Efficiency z-score for mothers who received IFA and MMN overall and for each subgroup. Mean *z*-scores are adjusted for cluster randomization, education, age, socio-economic index, and set of tests. Error bars show the standard error of the mean.

We followed up on the significant effect of MMN on the Reading Efficiency test by further examining this test in the undernourished and anemic subgroups. Among the 141 participants for whom mid-upper arm circumference at enrollment and reading scores were known, only those who were undernourished showed a significant benefit of MMN on the test (Table 6). Among the 153 participants for whom hemoglobin concentration at enrollment was known, only participants who were anemic showed a significant benefit of MMN on the reading test (Table 6). See Figure 4.

**Table 6:** The Effect of MMN on the Reading Efficiency test Z-Scores.

	Adjusted for Cluster Randomization			Adjusted for Cluster Randomization, set of tests, and maternal education, age, and socio-economic status		
	*n*	Estimate (95% CI)	*p*	*n*	Estimate (95% CI)	*p*
All participants	426	0.20 (0.01–0.39)	0.040	359	0.18 (0.02–0.35)	0.031
Mid-upper arm curcumference <23.5 cm	47	0.95 (0.41–1.48)	0.001	47	0.65 (0.19–1.12)	0.007
Mid-upper arm circumference > = 23.5 cm	94	0.00 (−0.39–0.39)	0.996	94	0.18 (−0.16–0.52)	0.300
Hemoglobin <110 g/L	77	0.54 (0.11–0.97)	0.016	76	0.40 (0.02–0.77)	0.039
Hemoglobin > = 110 g/L	76	0.01 (−0.42–0.45)	0.949	73	0.16 (−0.21–0.53)	0.395

*Note*. The estimate of the effect of MMN represents the estimate of the difference in reading scores between participants who received IFA and MMN, expressed as a fraction of the variation (standard deviation) of the Reading Efficiency test *z*-score.

These results suggest that reading efficiency was particularly sensitive to supplementation with multiple micronutrients and, just as for overall cognition, participants who showed signs of undernutrition and anemia benefited particularly in reading efficiency from supplementation with MMN.

## Discussion

In summary, this randomized trial examining the effects of maternal MMN supplementation as compared to IFA demonstrated benefits of MMN on overall maternal cognition, and on reading efficiency in particular. Mothers who were undernourished or anemic demonstrated particular benefits. Controlling for age, education, and socio-economic status, the increase in cognition due to MMN (*B* = 0.12; Table 4) was roughly equivalent to the increase in cognition with one year of education (*B* = 0.11). Among anemic women, the increase in cognition due to MMN (*B* = 0.20) was roughly equivalent to two years of education (2*0.11 = 0.22), and among the undernourished women (*B* = 0.33), to three years of education (3*0.11 = 0.33). No effects of MMN were found on maternal mood.

This is the first study showing a benefit of MMN supplementation on cognition during pregnancy and postpartum. Only two previous studies have examined the effect of micronutrient supplementation on cognitive outcomes in pregnant or postpartum women, and only one of these was conducted in a developing country. Both studies focused on the effects of supplementary iron and both were limited to small sample sizes. Groner, Holtzman, Charney, and Mellits [29] found that 16 American inner-city pregnant teenagers who received one month of supplementation with iron and vitamins significantly improved in tests of attention and declarative memory, while nine who received vitamins without iron did not improve on any test scores. A second study examined postpartum women in a poor peri-urban South African community. After six months, 30 anemic mothers who received folate and vitamin C with iron significantly improved in tests of reasoning and attention, while 21 anemic mothers who received folate and vitamin C without iron did not improve on either test [30]. Here, we have shown that in a larger sample of mothers MMN supplementation resulted in further cognitive benefits over and above any benefit of IFA, suggesting that micronutrients other than iron and folic acid are also critical for maternal cognition.

Although the present study was limited to pregnant and postpartum women in Lombok, the cognitive benefits of MMN might extend to other populations as well, especially elderly adults and children, who have been the focus of most previous research investigating micronutrients and cognition. Studies in these populations have reported positive concurrent associations between micronutrient status and overall cognitive function, [31], [32] as well as many of the specific abilities tested in the present study [33], [34]. However, the results of randomized trials have been inconsistent. Two recent systematic reviews of randomized trials of multiple micronutrient supplementation in adults age 65 and over [35] and children age 0–18 [36] each found little evidence for cognitive benefits of MMN and concluded that additional methodologically sound large scale trials are needed. Given the findings here, in future studies, it may be important to examine subgroups of participants who are undernourished or anemic in order to detect substantial effects on cognition. In the meta-analysis by Eliander, Gera, Sachdev et al. [36] the pooled estimate for the effect of MMN on fluid intelligence in children age 5–16 was 0.14 *SD* (95% CI: −0.02, 0.29; *p* = 0.083), which is almost identical to the effect of MMN on overall cognition found here (0.12 *SD*), suggesting that an effect of this magnitude may be expected even without focusing on subgroups of undernourished or anemic individuals.

In contrast to its positive effects on cognition, MMN showed no evidence of improving maternal motor dexterity or mood scores. In fact, mood scores were non-significantly lower in the MMN group than the IFA group, suggesting that enhanced mood cannot account for the positive effects of MMN on cognition. More generally, the finding that MMN did not enhance mood contributes to the literature examining the effects of maternal micronutrient supplementation on mood, in particular because the null effect observed here was found in the context of significant positive effects on cognition. Although the pattern reported here is different from two studies that found fewer depressive symptoms with maternal MMN supplementation [37], [38], it is consistent with the results of another study that did not find effects of maternal iron supplementation on postpartum depression, anxiety, or stress [30]. It remains to be seen whether future studies also suggest that maternal MMN supplementation fails to benefit mood during pregnancy or to alleviate postpartum depression.

The pattern of the benefit of MMN on cognition across all participants and in subgroups of anemic and undernourished mothers is consistent with the pattern of effects on early infant mortality reported by the SUMMIT Study Group [2]. Early infant mortality was reduced by 18% with maternal MMN supplementation over all mothers, by 38% for anemic mothers, and by 25% for undernourished mothers. Similarly, cognition was improved by the equivalent of one year of education over all mothers, two years of education for anemic mothers, and three years of education for undernourished mothers. These findings are consistent with the idea that improvements in infant survival may be partly mediated by improvements in maternal cognition.

The potential effect of improved maternal cognition on infant survival can be inferred from the relationship between maternal education and cognition reported here and the relationship between maternal education and infant mortality reported in other studies. Based on data from 175 countries, every one-year increase in education among women of reproductive age from 1970 to 2009 was associated with a reduction in infant mortality of 9.5% [39]. One mechanism through which maternal education may improve child health is through improving maternal cognition [40]. Since the improvement in maternal cognition with MMN supplementation observed here was equivalent to one year of education, it is plausible that up to 9.5% of the 18% reduction in infant mortality with MMN was due to improvement in maternal cognition. Likewise, since the effect of MMN on cognition was equivalent to two and three years of education for anemic and undernourished mothers, respectively, up to 19% of the 38% reduction in mortality in infants of anemic mothers might be attributed to improvements in maternal cognition, and possibly all of the 25% reduction in undernourished mothers. However, these numbers remain highly speculative. Moreover, factors other than cognition may also play a part in the reduction in infant mortality with maternal education, such as increased use of health services and increased maternal autonomy and input into family decisions in higher educated mothers [40], [41].

Further studies are needed to clarify the links between maternal interventions, maternal cognition, specific caregiving behaviors, and infant mortality, as well as other aspects of child health. For example, the benefit of maternal MMN supplementation on cognition reported here may partly mediate the effects of maternal MMN supplementation on subsequent physical growth (weight, head circumference, and mid-upper arm circumference) that have been observed at age 2.5 years [42] and the improvements in subsequent motor and mental development that have been observed in infants of mothers given MMN [43], [44], [45]. Several lines of evidence suggest that this is an important and promising area of study.

First, improving cognition in pregnant women seems particularly desirable given that this is a crucial period for acquiring knowledge and skills that are needed for child health and survival, such as health-seeking behaviors and child-care practices, and that many women experience cognitive changes during pregnancy. Subjective experiences such as increased absentmindedness and forgetfulness during pregnancy have been consistently reported [46], [47], [48], [49], [50]. Deficits in pregnant or postpartum women compared to non-pregnant controls on tests of cognition, including tests of declarative memory and verbal fluency, have also been reported [49], [51], [52], though not in all studies [50], [53], [54], [55], [56]. Cognitive changes during pregnancy may be explained by several mechanisms, including changes in hormone levels [57], mood alterations [58], and psychological adjustment to a major life change, which may interfere with normal cognitive processing [55]. Independent of the mechanisms of cognitive changes during pregnancy, improving cognition during this period through MMN supplementation or other means may facilitate memory of new health information and acquisition of caregiving skills.

Second, the benefit of maternal MMN supplementation on cognition reported here may extend to other maternal interventions as well. Two studies (discussed above) have shown improvements in cognition (attention, declarative memory, and reasoning) with iron supplementation during pregnancy or postpartum [29], [30]. Treating parasitic infection during pregnancy and treating or preventing malaria may also improve maternal cognition, since both parasitic infection and malaria are associated with cognitive deficits in school children [59], [60], [61], although the cognitive effects of these interventions have not been studied during pregnancy and postpartum. Therefore, several maternal interventions that are known to improve child health are also likely to improve maternal cognition.

Third, supplementation with certain micronutrients may be more effective before rather than after conception. For example, folic acid supplementation before but not after conception prevents neural tube defects [62] and iron supplementation before pregnancy has been found to improve maternal iron stores more than supplementation after pregnancy [63]. Our findings suggest that supplementation with multiple micronutrients during pre-conception care may confer the additional benefit of improving cognition in the mother as well as preparing micronutrient stores for the pregnancy.

Finally, maternal caregiving behavior is related to maternal intelligence, education, nutritional status, and health all of which are related to each other [10], [11], [64]. The UNICEF extended model of care specifies six categories of caregiver resources: knowledge and beliefs; health, nutritional status, and anemia; mental health and stress; control of resources and autonomy; workload and time constraints; and social support [65]. According to the model, these caregiver resources interact with economic resources and health resources to determine caregiving behavior which in turn influences child survival, growth, and development. Very few studies have attempted to clarify the extent to which various caregiver resources interact with and affect each other in their influence on child health. The finding in the current study that maternal MMN supplementation improved maternal cognition suggests that such models can be further specified and empirically tested to advance our understanding of the effects of caregiver resources on child health and the mechanisms of the effects of maternal interventions.

## Supporting Information

Protocol S1 **Trial Protocol.**
(PDF)

Checklist S1 **CONSORT Checklist.**
(DOC)

Appendix S1 **The contents of the two maternal supplements.**
(DOC)

## References

[1] Fall CHD, Fisher DJ, Osmond C, Margetts BM, the Maternal Micronutrient Supplementation Study Group (MMSSG) (2009). Multiple micronutrient supplementation duirng pregnancy in low-income countries: A meta-analysis of effects on birth size and length of gestation.. Food Nutr Bull.

[2] SUMMIT Study Group (2008). Effect of maternal multiple micronutrient supplementation on fetal loss and infant death in Indonesia: a double-blind cluster-randomised trial.. Lancet.

[3] Bicego GT, Boerma JT (1993). Maternal education and child survival: a comparative study of survey data from 17 countries.. Social Science & Medicine.

[4] Charmarbagwala R, Ranger M, Waddington H, White H (2004). The determinants of child health and nutrition: a meta-analysis.. The World Bank and the University of Maryland.

[5] Ceci SJ (1991). How much does schooling influence general intelligence and its cognitive components? A reassessment of the evidence.. Developmental Psychology.

[6] Sandiford P, Cassel J, Sanchez G, Coldham C (1997). Does intelligence account for the link between maternal literacy and child survival?. Social Science & Medicine.

[7] Anoop S, Saravanan B, Joseph A, Cherian A, Jacob KS (2004). Maternal depression and low maternal intelligence as risk factors for malnutrition in children: a community based case-control study from South India.. Archives of Disease in Childhood.

[8] Bhargava A (1999). Modeling the effects of nutritional and socioeconomic factors on growth and morbidity of Kenyan school children.. American Journal of Human Biology.

[9] Rubalcava LN, Terue GM (2004). The role of maternal cognitive ability on child health.. Economics and Human Biology.

[10] Jacobson S, Jacobson J, Frye K (1991). Incidence and correlates of breast feeding in socioeconomically disadvantaged women.. Pediatrics.

[11] Wachs TD, Creed-Kanashiro H, Cueto S, Jacoby E (2005). Maternal education and intelligence predict offspring diet and nutritional status.. Journal of Nutrition.

[12] Wachs TD, McCabe G (2001). Relation of maternal intelligence and schooling to offspring nutritional intake.. International Journal of Behavioral Development.

[13] Bhargava A, Fox-Kean M (2003). The effects of maternal education versus cognitive test scores on child nutrition in Kenya.. Econ Hum Biol.

[14] Galler JR, Harrison RH, Biggs MA, Ramsey F, Forde V (1999). Maternal moods predict breast-feeding in Barbados.. Journal of Developmental & Behavioral Pediatrics.

[15] Murray L, Cooper PJ, Wilson A, Romaniuk H (2003). Controlled trial of the short- and long-term effect of psychological treatment of post-partum depression.. British Journal of Psychiatry.

[16] Fredericksen CJ, Suh SW, Silva D, Frederickson CJ, Thompson RB (2000). Importance of zinc in the central nervous system the zinc containing neuron.. Journal of Nutrition.

[17] Squire LR, Stark CE, Clark RE (2004). The medial temporal lobe.. Annual Review of Neuroscience.

[18] Ullman MT (2004). Contributions of memory circuits to language: The declarative/procedural model.. Cognition.

[19] Beard JL, Connor JR (2003). Iron status and neural functioning.. Annual Review of Nutrition.

[20] Mackey AD, Davis SR, Gregory JF, Shils M, Shike M, Ross AC, Caballero B, Cousins RJ (2006). Vitamin B6.. Modern nutrition in health and disease. 10th ed.

[21] Rosenberg IH (2001). B vitamins, homocysteine, and neurocognitive function.. Nutrition Reviews.

[22] Nieoullon A (2002). Dopamine and the regulation of cognition and attention.. Prog Neurobiol.

[23] Willingham DB (1998). A neuropsychological theory of motor skill learning.. Psychological Review.

[24] Stahl SM (2000). Essential psychopharmacology. Neuroscientific basis and practical applications.

[25] Prado EL, Hartini S, Rahmawati A, Ismayani E, Hidayati A (2010). Test selection, adaptation, and evaluation: Three critical steps to assess nutritional influences on child development in developing countries.. British Journal of Educational Psychology.

[26] Wears RL (2002). Advanced statistics: Statistical methods for analyzing cluster and cluster-randomized data.. Acad Emerg Med.

[27] Satterthwaite FE (1946). An approximate distribution of estimates of variance components.. Biometrics Bulletin.

[28] Baayen HR (2004). Statistics in Psycholinguistics: A critique of some current gold standards.. Mental Lexicon Working Papers 1.

[29] Groner JA, Holtzman NA, Charney E, Mellits DE (1986). A randomized trial of oral iron on tests of short-term memory and attention span in young pregnant women.. J Adolesc Health Care.

[30] Beard JL, Hendricks MK, Perez EM, Murray-Kolb LE, Berg A (2005). Maternal iron deficiency anemia affects postpartum emotions and cognition.. Journal of Nutrition.

[31] Selhub J, Bagley LC, Miller J, Rosenberg IH (2000). B vitamins, homocysteine, and neurocognitive function in the elderly.. American Journal of Clinical Nutrition.

[32] Lozoff B (2007). Iron deficiency and child development.. Food Nutr Bull.

[33] Robins Wahlin TB, Wahlin A, Winblad B, Backman L (2001). The influence of serum vitamin B12 and folate status on cognitive functioning in very old age.. Biol Psychol.

[34] Louwman MW, van Dusseldorp M, van de Vijver FJ, Thomas CM, Schneede J (2000). Signs of impaired cognitive function in adolescents with marginal cobalamin status.. American Journal of Clinical Nutrition.

[35] Jia X, McNeill G, Avenell A (2008). Does taking vitamin, mineral, and fatty acid supplements prevent cognitive decline? A systematic review of randomized controlled trials.. Journal of Human Nutrition and Dietetics.

[36] Eilander A, Gera T, Sachdev HS, Transler C, van der Knaap HCM (2010). Multiple micronutrient supplementation for improving cognitive performance in children: Systematic review of randomized controlled trials.. American Journal Clinical Nutrition.

[37] Smith Fawzi MC, Kaaya SF, Mbwambo J, Msamanga GI, Antelman G (2007). Multivitamin supplementation in HIV-positive pregnant women: impact on depression and quality of life in a resource-poor setting.. HIV Medicine.

[38] Frith AL, Naved RT, Ekstrom E-C, Rasmussen KM, Frongillo EA (2009). Micronutrient supplementation affects maternal-infant feeding interactions and maternal distress in Bangladesh.. American Journal of Clinical Nutrition.

[39] Gakidou E, Cowling K, Lozano R, Murray CJ (2010). Increased educational attainment and its effect on child mortality in 175 countries between 1970 and 2009: a systematic analysis.. Lancet.

[40] Wachs TD (2008). Mechanisms linking parental education and stunting.. Lancet.

[41] Cleland JG, Van Ginnekan J (1988). Maternal education and child survival in developing countries: the search for pathways of influence.. Social Science & Medicine.

[42] Vaidya A, Saville N, Shrestha BP, de L Costello AM, Manandhar DS (2008). Effects of antenatal multiple micronutrient supplementation on children's weight and size at 2 years of age in Nepal: follow-up of a double-blind randomised controlled trial.. Lancet.

[43] McGrath N, Bellinger D, Robins J, Msamanga GI, Tronick E (2006). Effect of maternal multivitamin supplementation on the mental and psychomotor development of children who are born to HIV-1-infected mothers in Tanzania.. Pediatrics.

[44] Tofail F, Persson LA, Arifeen SE, Hamadani JD, Mehrin F (2008). Effects of prenatal food and micronutrient supplementation on infant development: a randomized trial from the Maternal and Infant Nutrition Interventions, Matlab (MINIMat) study.. American Journal of Clinical Nutrition.

[45] Li Q, Yan H, Zeng L, Cheng Y, Liang W (2009). Effects of maternal micronutrient supplementation on the mental development of infants in rural western China: Follow-up evaluation of a double-blind, randomized, controlled trial.. Pediatrics.

[46] Casey P (2000). A longitudinal study of cognitive performance during pregnancy and new motherhood.. Archives of Women's Mental Health.

[47] Brindle PM, Brown MW, Brown J, Griffith HB, Turner GM (1991). Objective and subjective memory impairment in pregnancy.. Psychological Medicine.

[48] Poser CM, Kassirer MR, Peyser JM (1986). Benign encephalopathy of pregnancy: Preliminary clinical observations.. Acta Neurologica Scandinavia.

[49] Sharp K, Brindle PM, Brown MW, Turner GM (1993). Memory loss during pregnancy.. British Journal of Obstetrics and Gynaecology.

[50] McDowell J, Moriarty R (2000). Implicit and explicit memory in pregnant women: An analysis of data-driven and conceptually driven processes.. The Quarterly Journal of Experimental Psychology.

[51] DeGroot RHM, Hornstra G, Roozendaal N, Jolles J (2003). Memory performance, but not information processing speed, may be reduced during early pregnancy.. Journal of Clinical and Experimental Neuropsychology.

[52] DeGroot RHM, Vuurman EFPM, Hornstra G, Jolles J (2006). Differences in cognitive performance during pregnancy and early motherhood.. Psychological Medicine.

[53] Casey P, Huntsdale C, Angus G, James C (1999). Memory in pregnancy. II: Implicit, incidental, explicit, semantic, short-term working, and prospective memory in primigravid, multigravid and postpartum women.. Journal of Psychosomatic Obstetric Gynaecology.

[54] Christensen H, Poyser C, Pollitt P, Cubis J (1999). Pregnancy may confer a selective cognitive advantage.. Journal of Reproductive and Infant Psychology.

[55] Crawley RA, Dennison K, Carter C (2003). Cognition in pregnancy and the first year post-partum.. Psychology and Psychotherapy: Theory, Research and Practoce.

[56] Christensen H, Leach LS, Mackinnon A (2010). Cognition in pregnancy and motherhood: prospective cohort study.. British Journal of Psychiatry.

[57] Buckwalter JG, Buckwalter DK, Bluestein BW, Stanczyk FZ (2001). Pregnancy and post partum: Changes in cognition and mood.. Prog Brain Res.

[58] Evans J, Heron J, Francomb H, Oke S, Golding J (2001). Cohort study of depressed mood during pregnancy and after childbirth.. British Medical Journal.

[59] Ezeamama AE, Friedman JF, Acosta LP, Bellinger DC, Langdon GC (2005). Helminth infection and cognitive impairment among Filipino children.. Am J Trop Med Hyg.

[60] Jukes MC, Nokes CA, Alcock KJ, Lambo JK, Kihamia C (2002). Heavy schistosomiasis associated with poor short-term memory and slower reaction times in Tanzanian schoolchildren.. Trop Med Int Health.

[61] Holding PA, Snow RW (2001). Impact of *Plasmodium Falciparum* malaria on performance and learning: Review of the evidence.. American Journal of Tropical Medicine and Hygiene.

[62] Pitkin RM (2007). Folate and neural tube defects.. The American journal of clinical nutrition.

[63] Milman N, Bergholt T, Byg KE, Eriksen L, Graudal N (1999). Iron status and iron balance during pregnancy. A critical reappraisal of iron supplementation.. Acta obstetricia et gynecologica Scandinavica.

[64] Winkvist A (1995). Health and nutrition status of the caregiver: Effect on caregiving capacity.. Food and Nutrition Bulletin.

[65] Engle PL, Menon P, Garrett J, Slack A (1997). Urbanization and caregiving: A framework for analysis and examples from southern and eastern Africa.. Environ Urban.

[66] Rey A (1964). L'examen Clinique en Psychologie.

[67] Wechsler D (1997). Wechsler Adult Intelligence Scale III.

[68] Lezak MD (2004). Neuropsychological assessment.

[69] Sakurai Y, Takeuchi S, Kojima E, Yazawa I, Murayama S (1998). Mechanism of short-term memory and repetition in conduction aphasia and related cognitive disorders: a neuropsychological, audiological and neuroimaging study.. Journal of Neurological Sciences.

[70] Silveri MC, Di Betta AM, Filippini V, Leggio MG, Molinari M (1998). Verbal short-term store-rehearsal system and the cerebellum. Evidence from a patient with a right cerebellar lesion.. Brain.

[71] Desmond JE, Fiez JA (1998). Neuroimaging studies of the cerebellum: language, learning, and memory.. Trends in Cognitive Sciences.

[72] Smith EE, Jonides J (1998). Neuroimaging analyses of human working memory.. Proceedings of the National Academy of Sciences of the United States of America.

[73] Ekstrom RB, French JW, Harman HH, Dermen D (1976). Manual for Kit of Factor-referenced Cognitive Tests.

[74] Richter W, Somorjai R, Summers R, Jarmasz M, Menon RS (2000). Motor area activity during mental rotation studied by time-resolved single-trial fMRI.. Journal of Cognitive Neuroscience.

[75] Jordan K, Heinze HJ, Lutz K, Kanowski M, Jancke L (2001). Cortical activations during the mental rotation of different visual objects.. NeuroImage.

[76] Obonswain MC, Crawford JR, Page J, Chalmers P, Cochrane R (2002). Performance on tests of frontal lobe function reflect general intellectual ability.. Neuropsychologia.

[77] Misra M, Katzir T, Wolf M, Poldrack RA (2004). Neural systems for rapid automatized naming in skilled readers: Unraveling the RAN-reading relationship.. Scientific Studies of Reading.

[78] Torgesen JK, Wagner R, Rashotte C (1999). Test of Word Reading Efficiency (TOWRE).

[79] Fiez J, Petersen S (1998). Neuroimaging studies of word reading.. Proceedings of the National Academy of Science.

[80] Fiez JA, Balota DA, Raichle ME, Petersen SE (1999). Effects of lexicality, frequency, and spelling-to-sound consistency on the functional anatomy of reading.. Neuron.

[81] Biswal B, Ulmer JL, Krippendorf RL, Harsch HH, Daniels DL (1998). Abnormal cerebral activation associated with a motor task in Tourette syndrome.. American Journal of Neuroradiology.

[82] Lemon RN (1999). Neural control of dexterity: what has been achieved?. Experimental Brain Research.

[83] Radloff LS (1977). The CES-D Scale: A self-report depression scale for research in the general population.. Applied Psychological Measurement.

[84] Pinel JPJ (2006). Biopsychology.

[85] Hariri AR, Mattay VS, Tessitore A, Kolachana B, Fera F (2002). Serotonin transporter genetic variation and the response of the human amygdala.. Science.

