# Supplementary material for: The Effect of Maternal Multiple Micronutrient Supplementation on Cognition and Mood during Pregnancy and Postpartum in Indonesia: A Randomized Trial
Source: PLoS One. 2012 Mar 12;7(3):e32519. doi: 10.1371/journal.pone.0032519 (PMC3299672; doi:10.1371/journal.pone.0032519)
Supplement: Protocol S1 — Trial Protocol. (PDF) [file pone.0032519.s001.pdf]

Appendix A

Five-year Activity Proposal

Supplementation with Multiple Micronutrients Intervention Trial (SUMMIT)

A Prenatal Intervention to Reduce Maternal Mortality and Improve Infant Health

Submitted to:

USAID/I/PHN and the
Ministry of Health of the Republic of Indonesia,
Jakarta, Indonesia

By:

HELEN KELLER INTERNATIONAL/Indonesia
May 8, 2000

The Maternal SUMMIT Activity:

SUpplementation with **M**ultiple **M**icronutrients **I**n**T**ervention to
Reduce Maternal Mortality and Improve Infant Health

Executive Summary

Introduction

Maternal mortality, low birth weight, and early infant mortality remain important public health problems in most developing countries. Although it is widely acknowledged that poor prenatal nutritional status in developing countries adversely affects maternal and infant health, prenatal nutrient supplementation is generally limited to the provision of iron/folate tablets. This contrasts sharply to the prevailing situation in developed countries, where long-standing policies of routine antenatal care include multi-vitamin/mineral supplements.

The potential importance of prenatal multinutrient supplements is demonstrated by several studies in developing countries which documented substantial improvements in maternal and infant health following supplementation with individual nutrients or combinations of nutrients including vitamin A, iron, zinc, and folate. Indeed, one recent report from Nepal found that routine supplementation of women with vitamin A (retinol) or β-carotene, alone, reduced pregnancy-related mortality by 40%. Prenatal multinutrient supplementation is, therefore, strongly indicated and further underscored by the high overall prevalence of micronutrient deficiencies in developing countries.

The encouraging effects of prenatal nutrient supplementation on maternal and infant health have generally been obtained under somewhat artificial highly supervised trial conditions. Before clear policy decisions can be made regarding the provision of prenatal multi-micronutrient supplements, it is also necessary to determine the health benefits of multinutrients when delivered through existing programs of prenatal care.

Specific Aims

Helen Keller International (HKI)-Indonesia, therefore, proposes to conduct a **Su**pplementation with **M**ultiple **M**icronutrients **Int**ervention (SUMMIT) to determine the health impact of pre-natal multiple micronutrient distribution through the existing health infrastructure. The primary purpose of the intervention is to determine if daily prenatal multi-micronutrient supplementation will:

1. Reduce the frequency of maternal mortality
2. Reduce the proportion of low birth weight neonates
3. Reduce neonatal and infant mortality rates

Intervention area and population

The proposed intervention site is the tropical island of Lombok, NTB, which remains one of the poorest regions of the country with correspondingly poor health indicators. Infant and under-five mortality per 1000 live births are 110 and 149, respectively, with an estimated maternal mortality ratio ranging from 400 to 800 per 100,000 live births. The island occupies approximately 4,739 km^2 and is home to 2.6 million people. At 568 persons/km^2 it is one of the most densely populated regions in Indonesia.

Reduction of maternal and infant mortality are priorities of the Ministry of Health (MOH) of Indonesia. In the last decade several maternal health promotion activities have been implemented through the MOH, including placement of trained midwives in the majority of villages, and education programs to improve maternal health before and during pregnancy. Thus, the Indonesian health structure on Lombok is well positioned to assess the programmatic impact of prenatal multinutrient supplementation on maternal mortality, low birth weight, and infant health.

SUMMIT activity design and methodology

Outcomes: The SUMMIT activity is a controlled trial designed to assess the relative impact of a prenatal multi-micronutrient tablet in comparison to the current MOH iron/folate tablet on frequency of maternal mortality as assessed by both the maternal mortality ratio (MMR), defined as maternal deaths per 100,000 live births, and the maternal mortality rate (MMRT), defined as the death of a woman while pregnant or within 42 days of termination of pregnancy from any cause aggravated or related to the pregnancy, expressed per 100,000 pregnancies. Two additional main outcomes of the SUMMIT activity are proportion of low birth weight infants, and infant mortality, including neonatal mortality. Secondary outcomes to be assessed include maternal morbidity, neonatal development, and nutritional status during pregnancy and lactation. In addition to examining the effect of multi-nutrients, we will document the effect of education and social marketing of the SUMMIT activity by comparing outcome rates in the iron/folate arm of the intervention area to corresponding rates in designated villages where standard prenatal care practice will continue. In a 20% subsample of pregnancies biochemical status for micronutrients, including, iron, zinc, and vitamin A, will be determined.

Sample Size: The SUMMIT activity will aim to detect a reduction in both MMR and MMRT of at least 30% with a power of 80% and significance level of 0.05. Current estimates of the MMR in various districts on Lombok range from 400 to 800 maternal deaths per 100,000 live births and randomization for the trial will be by clusters consisting of geographic areas serviced by each of the 300 midwives providing prenatal care. Assuming 400 maternal deaths per 100,000 live births and a 10% loss to follow-up, the trial would require enrollment of 102,000 live birth pregnancies allocated to either an iron/folate or multinutrient supplement to detect a 30% reduction in MMR. Because not all pregnancies result in a live birth, it is estimated that the required sample size be increased an additional 15% to 120,000. This sample size will also allow detection of similar reductions in MMRT. It is proposed that enrollment be phased in over a 6-month period and continue for at least 2 years to accommodate seasonal effects. The phased in implementation along with the approximately 72,000 pregnancies occurring each year throughout the trial area yields a projected enrollment of 126,000 pregnancies. The additional margin of enrollment provides the ability to detect reductions in MMR as little as 22% if ratios approach 800 per 100,000 live births. With regard to low birth weight and infant mortality rates, the projected sample size will allow detection of at least a 10% reduction in low birth weight and infant mortality rates, including neonatal mortality rates. Equally important is determination of the impact of the social marketing and education programs that will accompany the trial implementation in the intervention area. Data concerning outcome variables in the absence of the intervention will be collected during the phase-in period prior to enrollment. This will result in the ability to detect at least a 25% difference in MMR between the iron/folate arm of the intervention area compared non-intervention areas.

Supplementation: Pregnant women will be provided with either an iron and folate tablet or another multi-micronutrient tablet containing iron, folate, vitamins A, B1, B2, B6, B12, C, D, and E, along with niacin, zinc, copper, selenium, and iodine. Supplements will be provided by existing MOH midwives, and be distributed from the first antenatal consultation for a confirmed pregnancy until three months postpartum. The midwives will distribute the supplements monthly as 30-tablet sachets, as is current MOH practice, and monitor the health impact through routine prenatal care visits and compliance checks. Additional compliance monitoring will be conducted by HKI field workers through spot-checking of pill counts and biochemical indices.

Monitoring: In addition to the distribution of supplements, midwives will continue to record the results of routine prenatal examinations, attend deliveries and record data on labor and delivery, birth weight, and APGAR scores of neonates. All of these activities are currently part of the normal maternal care protocol implemented by the MOH. The implementation of these activities will be strengthened in all villages by training and community promotion. HKI field workers will collect additional data at enrollment, at 36 weeks of gestation, and at 12 weeks postpartum on socio-economic status, food intake, and anthropometry. Deaths will be detected through the longitudinal surveillance of participants and also through the quarterly island-wide demographic surveillance system. The dual surveillance will allow the trial to document all maternal deaths (up to and exceeding 12 weeks postpartum), to allow evaluation of MMR and MMRT, and all infant deaths occurring beyond the 12-week visit. Lastly, it will allow monitoring of mortality in the non-intervention villages. For all maternal and infant deaths verbal post-mortem assessments will be made to determine the associated causes and conditions.

Implementation schedule: The trial will be carried out over five years in 3 distinct stages broadly scheduled as follows; Phase I- Preparation and training from December 1999-August 2000, Phase II-Supplementation and follow-up from September 2000-September 2003, and Phase III-Analysis and report preparation from October 2003-October 2004.

Relevance to MOH and USAID objectives

The proposed maternal SUMMIT activity is directly in line with the USAID/I Mission's Strategic Objective, "Protecting the Health of the Most Vulnerable Women and Children". It also fully supports USAID/I's "Essential Services Component Two: Maternal and Neonatal Health (MNH)," by working through the existing health care system to assist in "maintaining and ultimately improving the quality and delivery of services that have proven effective in addressing the most important causes of maternal and neonatal morbidity and mortality." SUMMIT contributes directly to the Essential Package of Safe Motherhood Services, focused on improving the clinical skills of midwives for antenatal care and birthing practice for safe delivery; management of complications and timely referrals; identification of risk factors for neonatal mortality; care of normal and sick neonates; and postpartum care. It also strengthens "demand generation and community empowerment efforts to improve birth preparedness," and "the technical and management capacity" of midwives, and includes efforts to "improve the utilization of safe motherhood and child health services at the community level." SUMMIT also works toward "a decrease in the prevalence of anemia among pregnant women in project areas" (IR1: Essential Services Preserved) and an increase in the "percentage of adults knowledgeable about maternal complications of pregnancy and childbirth" (IR3: Promotion of Appropriate Behaviors and Services).

Appendix A

I. INTRODUCTION 3

A. Background 3

B. Problem 4

II. CAUSES OF MATERNAL MORTALITY AND LINKS TO NUTRITION 7

A. Hemorrhage and Anemia 7

B. Pre-eclampsia 8

C. Obstructed labor and prolonged labor 8

D. Infection 8

III. INDICATED BENEFITS OF MICRONUTRIENTS ON MATERNAL MORTALITY, BIRTH WEIGHT, AND INFANT MORTALITY 9

A. Vitamin A 9

B. Iron 10

C. Folate 11

D. Zinc 11

IV. CHOOSING AND EVALUATING A MICRONUTRIENT SUPPLEMENT FOR PREGNANT WOMEN 11

V. SUPPLEMENT DISTRIBUTION 12

VI. OBJECTIVES AND GOALS 13

A. General objectives/goals 13

B. Specific objectives 13

VII. PROJECT ACCOMPLISHMENTS TOWARD USAID/I MISSION'S STRATEGIC OBJECTIVES 13

Relationship of HKI with other Organizations and the Government of Indonesia 14

VIII. IMPLEMENTATION 14

A. Schedule 14

B. Implementation methods 15

1. Study area and population 15
2 . Design and Methods 16
3 . Sample Size 16
4 . Demographic Baseline Survey 18
5 . Enrollment of pregnancies 19
6 . Randomization to treatment 19
7 . Supplement distribution and community cooperation 20
8 . Data collection 20

9 . Biochemical determinations 21
10 . Verbal autopsies 22
11 . Supervision, compliance and quality control 22
12 . Data management 22
13 . Consent procedures and ethical approval 23

IX. RESULTS 25

A. Inputs 25

B. Anticipated Benchmarks 25

C. Outputs 25

D. Results 25

E. Proposed indicators 25

X. REFERENCES 26

XII. GLOSSARY 29

A. Definitions 29

B. Abbreviations and acronyms 30

XIII. APPENDICES 31

Appendix 1: Brief description of HKI 1-1
Appendix 2: Research site 2-1
Appendix 3: a) Composition of the SUMMIT Multi-Micronutrient Supplement 3-1
b) Proceedings of the UNICEF/WHO/UNU Workshop: 'Composition of a Multi-Micronutrient Supplement to be used in Pilot Programs among Pregnant Women in Developing Countries' 3-2
Appendix 4: SUMMIT Organizational chart 4-1
SUMMIT Committees 4-2
Appendix 5: Timeline of activities 5-1
Appendix 6: Ethical approval from Johns Hopkins University 6-1
Sample Consent Form 6-2

I. INTRODUCTION

A. Background

Maternal and infant mortality remains an important public health problem in most developing countries. Both the maternal mortality ratio and infant mortality rates in Indonesia are among the highest in Southeast Asia.[1] Despite great strides in reducing child mortality, reducing fertility and improving health status, rates for maternal, neonatal, and infant mortality remain remarkably high. There is a need for novel, cost-effective and targeted interventions to improve these health indicators. It is widely accepted that nutritional status, specifically micronutrient status, is one of the most important contributing factors to overall maternal health, birth outcome, and infant health. Indeed, the prevalence of micronutrient deficiencies remains high in developing countries, and pregnant and lactating women are among the most vulnerable because of their relatively high micronutrient requirements. Unfortunately, in most developing countries (where women's diets are marginal and health is poor) prenatal micronutrient supplementation is limited to the provision of iron/folate tablets. This contrasts sharply to the prevailing situation in developed countries (where women's diets and health are relatively good), where long-standing policies of routine antenatal care include multi-vitamin/mineral supplements.

The potential importance of poor prenatal micronutrient status is underscored by several studies in developing countries that demonstrate substantial benefits of micronutrient supplementation, including vitamin A, iron, zinc, and folate on maternal and infant health. The recent Nepal Nutrition Intervention Program-Sarlahi 2 (NNIPS-2) trial found that supplementation with vitamin A (retinol) or β-carotene among 45,000 women of reproductive age reduced pregnancy-related mortality by 40%.[2] In addition, there is evidence from a variety of studies suggesting that other micronutrients given during pregnancy and lactation may contribute to the reduction of maternal and infant mortality.[3] Health personnel worldwide are increasingly recognizing the positive impact of micronutrient supplementation on maternal and infant health,[4] and the potential benefit of health programs focused on improving the micronutrient status of women during pregnancy and lactation.[5]

Most of the above-mentioned nutrient intervention studies were aimed at measuring the impact of micronutrient supplements on maternal and infant health under rigorous conditions of supervised delivery and monitoring. To fully assess the programmatic potential of such interventions it is crucial to determine the *effectiveness* of micronutrient supplementation when delivered through existing antenatal care systems. The most informative setting to evaluate the programmatic effectiveness of pre-natal multi-micronutrient supplementation would include regions where strong government support exists for promoting maternal health. In this context, Indonesia provides an ideal venue to examine the impact of pre-natal multi-micronutrient supplementation on maternal and infant health.

Reduction of maternal mortality is a priority of the Ministry of Health (MOH) in Indonesia and a variety of program interventions have been implemented through the MOH Directorate General of Community Health and Directorate of Family Health. These are described in the *Strategies to Accelerate the Reduction of Maternal Mortality* and include a broad range of technical, management, and educational efforts intended to

improve obstetric services and referral systems, and to educate communities about maternal health issues.

The Indonesia Safe Motherhood initiative formally began in June 1988. This resulted in the "Healthy and Prosperous Mother Campaign" implemented in 1990 which included the training and placement of trained midwives (*bidan di desa*) in villages as a means to improve access to appropriate maternal care. These efforts were intended to be implemented through antenatal visits to the midwives and increased community awareness about the importance of maternal health for safe motherhood. With respect to nutritional status, the strategy included "efforts to improve mother's health status before/during pregnancy, such as control of anemia and chronic energy malnutrition".[6] In December 1996, the complementary "Mother Friendly Movement" (*Gerakan Sayang Ibu*) program was launched, due to concern over slower than expected progress towards reducing the maternal mortality ratio. The two key elements of this program, "Mother-Friendly Communities" and "Mother-Friendly Health Care Facilities", sought to link communities with health care facilities, in an effort to overcome a variety of interconnected obstacles, including poor access, lack of referrals, inadequate transportation, and low delivery attendance by trained personnel.

In early 1999, the MOH commissioned a review and recommendations exercise, conducted by JHPIEGO and supported by USAID, to suggest ways to further improve the current efforts to reduce maternal mortality. This renewed interest in developing policy and program interventions to reduce the high rate of maternal and also infant mortality in Indonesia will require a variety of innovative approaches and interventions, including effective preventive strategies that can be provided at the community level, such as multi-micronutrient supplementation.

Helen Keller International (HKI), therefore, proposes to carry out a **SU**pplementation with **M**ultiple **M**icronutrients **I**n**T**ervention (SUMMIT) activity targeted at improving maternal micronutrient status on the island of Lombok, Nusa Tenggara Barat (NTB) province, where maternal and infant health indicators rank among the poorest in Indonesia.

The specific goals of the SUMMIT activity are to determine if daily multiple micronutrient supplementation during the prenatal period through 3 months post delivery will:

I. Reduce the frequency of maternal mortality

II. Reduce the proportion of low birth weight newborns

III. Reduce neonatal and infant mortality rates

B. Problem

Indonesia's maternal mortality ratio (MMR) of 390 per 100,000 live births is one of the highest in Asia.[7] The MOH estimates that this is three to six times the ratio of other ASEAN countries, and more than 50 times that of developed countries.[7] In addition, women may experience acute obstetric problems,[8] and long-term complications of pregnancy and childbirth such as prolapse, fistulae, pelvic inflammatory disease,

incontinence and infertility.[9] The burden of prenatal infection and disease is also substantial.

Various studies have estimated provincial MMR ranging from 331 per 100,000 live births in Bali to more than 975 per 100,000 in Irian Jaya.[10] The poor health-related conditions resulting in high MMR for different provinces also result in high neonatal and infant mortality rates. The island of Lombok in NTB is recognized as having one of the highest rates of infant mortality in Indonesia with correspondingly poor indices of general health status. Although province-specific maternal mortality ratios are not yet firmly established in NTB, infant and under-five mortality rates are high (110.5 and 149.5 compared to 52 and 71 for Indonesia overall, respectively), strongly suggesting that maternal mortality ratio is also higher in this province. Table 1 shows a comparison of mortality ratios for Indonesia as a whole, and NTB province. For comparison, data from Nepal are also presented.

Table 1. Mortality per 1,000 live births in Indonesia, Nusa Tenggara Barat (NTB) province and Nepal

	Indonesia	Nusa Tenggara Barat [ii]	Nepal[i]
Infant mortality rate (IMR)	52.2 [ii]	110.5	82
Underfive mortality rate (U5MR)	70.6 [ii]	149.5	116
Maternal mortality ratio (MMR)	3.9 [i]	4.0-8.9 [iii]	7.2-15

[i] Estimated from *The State of the World's Children 1998*, UNICEF 1998; pp119-20 (Actual data from 1990) and NNIPS-2 study data[2]
[ii] Data from *Indonesia Demographic and Health Survey 1997.* Central Bureau of Statistics/National Family Planning Coordinating Board/Ministry of Health/Macro International Inc., September 1998.
[iii] Estimated from *Indonesia Demographic and Health Survey 1997.* Central Bureau of Statistics/National Family Planning Coordinating Board/Ministry of Health/Macro International Inc., September 1998.

Child and infant mortality rates in NTB Province clearly exceed those for the whole of Indonesia, and even for Nepal, one of the few Asian countries where the maternal mortality ratio is higher than Indonesia. Maternal mortality in the province of Lombok is, therefore, believed to be among the highest in Indonesia.

The economic, environmental and political crisis that has gripped Indonesia since July 1997 has increased the prevalence and severity of micronutrient deficiencies. The crisis has caused a 75% devaluation of the rupiah, enormous increases in prices of basic commodities, and widespread unemployment. In July 1998, Indonesia's Central Bureau of Statistics reported that the number of Indonesians living below the poverty line had reached 79.4 million, over 1/3 of the population. In any crisis situation, food expenditures are compromised. Data collected in different areas of Indonesia show that the consumption of foods of high nutritional quality, such as meat and animal products (i.e. eggs, liver), has decreased the most.[11] These foods are particularly rich in micronutrients, such as iron, zinc and vitamin A. Thus, meeting the relatively high nutritional demands of pregnancy has become even more difficult.

Several studies have found deficiencies of iron and vitamin A among women of reproductive age in Java.[12,13,14,15] Among pregnant women, the prevalence of anemia is approximately 50%. An intervention trial among pregnant women found that combined supplementation of iron and vitamin A resulted in a larger reduction of the prevalence of anemia than iron alone, indicating that sub-clinical vitamin A deficiency is also a problem

among pregnant women.[12] When the intake of iron and vitamin A is relatively low, the intake of other micronutrients such as zinc and B-vitamins is likely to be low as well. In the January 1999 data collection round, the HKI Nutrition Surveillance System found that among non-pregnant women in Lombok the prevalence of wasting was 17.7%; nightblindness, 1.8%; and anemia, 28.6%. It is expected that the micronutrient status of pregnant women may be even lower.

Pregnant women who live in rural areas are young, have high birth orders, and/or no education are less likely to be assisted during their deliveries by trained medical staff than other women.[10] Virtually all assessments of the maternal health problem in Indonesia recognize that one of the key underlying problems associated with high maternal mortality is that four out of five women deliver their babies at home, often unattended by a trained provider.[16] In 1997, only 21% of births were attended by a government-trained midwife or physician in NTB province, well below the national average of 43% and the lowest rate of all provinces in that year.[16] It is also known that, once an obstetric emergency happens, many women die due to late arrival at health facilities or find that appropriate obstetric services are unavailable at the facility. The MOH recognizes that much of the country's maternal mortality can be attributed to three types of "delays": (1) Delay in recognizing the need for referral and late decision-making to seek appropriate care; (2) Delay in reaching the health care facility; and (3) Delay in getting adequate care in the health facility.[17] The MOH has a goal of increasing to 60% the number of women whose deliveries are assisted or supervised by a trained provider.

This highlights not only the need to strengthen the current health care system and the skills of providers, but also the need for interventions that reduce the risk of an obstetric emergency. Thus, along with strengthening the health care system and improving health seeking behavior and assisted delivery, interventions aimed at prevention of obstetric complications associated with poor health, including poor nutritional status, would be desirable. Indeed, maternal nutrition has been identified by the MOH as an important factor contributing to high maternal mortality in Indonesia.

As previously mentioned, Indonesia has placed trained midwives (*bidan di desa*) in the majority of the country's villages as a means to improve access to appropriate maternal care. These midwives receive 3 years of nurses' training followed by an additional 1 year of instruction in midwifery. The midwives serve as the front line workers for the MOH's maternal health program at the community level. They are responsible for providing antenatal care, screening for high-risk pregnancies, distributing iron/folate supplements, assisting with deliveries and referring women to appropriate health facilities for birth complications and postpartum care. The 1997 Indonesian Demographic and Health Survey (IDHS) found that women were eager to attend antenatal visits with the *bidan di desa* (with an average of 6.6 antenatal visits per live birth). This indicates the strong potential benefit of interventions delivered during prenatal contacts. Thus, if multiple micronutrient supplementation can be demonstrated to reduce maternal and infant mortality, sustainable delivery of this intervention through the trained midwives would be feasible.

II. CAUSES OF MATERNAL MORTALITY AND LINKS TO NUTRITION

As mentioned above, several factors contribute to maternal mortality, many of which are preventable. Approximately 60% of maternal mortality cases worldwide are the result of direct obstetric causes. These include: hemorrhage (25%), infection/sepsis (15%), eclampsia (12%), and obstructed labor (8%).[8] The 1995 National Household Survey reported the causes of maternal death in Indonesia as follows[18]:

Causes of Maternal Mortality in Indonesia	Percent of total maternal deaths
Hemorrhage	45.2
Eclampsia	12.0
Complications of Miscarriage	11.1
Postpartum sepsis	9.6
Prolonged Labor	6.5
Anemia	1.6
Other Indirect	14.1

Although the etiology of these causes is complex, available evidence indicates that many causes may be directly or indirectly influenced by micronutrient status.

A. Hemorrhage and Anemia

Hemorrhage, the most common cause of maternal death in Indonesia, is nearly double the global average and constitutes a serious problem for maternal health. As detailed below, anemia is a strong risk factor for hemorrhage-related death. However, high fatality rates from hemorrhage may also be strongly related to poor tissue integrity, leading to enhanced bleeding, and reduced thrombosis, both of which are exacerbated by micronutrient deficiencies such as zinc, copper, and B vitamins.

With respect to anemia, a review by Ross and Thomas of 21 studies in Africa and Asia estimated that 7-10% of maternal mortality is the direct result of anemia. In addition, another 10-15% of maternal deaths was indirectly related to anemia, mainly as a result of postpartum hemorrhage.[19] The potential burden of anemia on maternal and child health in Indonesia is underscored by the 1995 *Survei Kesehatan Rumah Tangga* (SKRT), which found that 50% of pregnant women, 30% of female workers, and 20-30% of female adolescents were anemic. A study by Chi et al. in Indonesia found that pregnant women with Hb < 10 g/dL were at 3 times higher risk of death than were women without anemia (Hb >11 g/dL).[20] Blood loss is a more severe problem for anemic women and a study in Bali[21] showed that 67% of maternal deaths were related to maternal hemorrhage. Moreover, anemia is associated with preterm delivery, perinatal mortality,[22,23] low birth weight,[24] and prolonged labor due to lethargy. Although iron deficiency is considered the most common cause of anemia, vitamin A deficiency is also implicated, as demonstrated by a study in Indonesian pregnant women wherein the largest reduction of anemia prevalence was achieved by supplementing them with both vitamin A and iron, as compared to either alone.[12]

Pre-eclampsia

Pre-eclampsia is a disorder solely related to pregnancy. The principal clinical symptoms are hypertension and proteinuria. Multiple organs become involved in this syndrome, which can cause renal and liver function disturbances, cerebral hemorrhage, and possibly death. Placental vascular changes result in decreased utero-placental blood flow, causing intra-uterine growth retardation and subsequent risk of perinatal morbidity and mortality.[25] Although the exact pathophysiology of pre-eclampsia is still unknown, local and/or systemic immunological reactions, endothelial damage, platelet activation, and low levels of calcium,[26] β-carotene[27] and retinol[27,28] may all play a role.

C. Prolonged labor and obstructed labor

Prolonged labor has many etiologies and results in failed or impaired transition from early to late stages of delivery. Dysregulated hormonal control of delivery-related physiological changes have been implicated amongst other causes. Obstructed labor is the consequence of mal-presentation of the fetus or cephalo-pelvic disproportion wherein the birth canal is unable to accommodate passage of the head of the fetus. The latter can result from a narrow birth canal of the pelvis and/or insufficient dilation of the cervix or elasticity of other soft tissues surrounding the birth canal. Improved pre-natal nutrition could potentially modulate both prolonged labor and obstructed labor by influencing the hormonal-mediated changes, including softening of the pelvic sutures, and elasticity of other soft tissues, resulting in greater opening of the birth canal. Moreover, vitamin A and zinc have been implicated as crucial nutrients in steroid hormone regulation and metabolism.

Short maternal height is a well-established risk factor for obstructed birth,[29] and is caused by intra-uterine growth retardation of the mother and/or poor growth during early childhood as a result of inadequate feeding and frequent infections. This was indicated by intervention studies in Guatemala, which found that an improvement of nutritional status during pregnancy resulted in larger babies, and a small improvement in nutritional status during early childhood resulted in taller adults.[30] Pelvic deformity, a consequence of rickets, may also contribute to obstructed birth. Improved nutrition of pregnant women may, therefore, have the long-term benefit of reducing obstructed labor in the next generation of women. Importantly, the notion that improved prenatal nutriture can result in increased rates of cephalo-pelvic disproportion has not been supported by data from interventions which improved prenatal nutritional status.[31]

D. Infection

Infection is a major cause of maternal mortality and poor neonatal/infant health. Infections may occur during pregnancy, delivery, and especially shortly after delivery. Both vitamin A and zinc have been shown to be essential to proper functioning of the immune system, and areas of the world where vitamin A and zinc deficiency are prevalent have higher maternal mortality ratios. There is increasing evidence of a positive relationship between vitamin A and zinc deficiency and the rate of infection.[30,32] In addition, work by Shankar *et al.* in Peru and Osendarp *et al*. in Bangladesh indicate that prenatal zinc supplementation decreased infant morbidity.[33]

In summary, there is a plausible link between micronutrient status and causes of maternal mortality. Moreover, there is evidence that poor maternal micronutrient status can lead to poor neonatal health, low birth weight, and increased infant morbidity and mortality. Indeed, research findings are increasingly pointing to the specific role of micronutrients in improving several maternal and child health indicators. An overview of such evidence is presented below, and provides further rationale to the notion that a prenatal multiple micronutrient supplementation is likely to optimize the potential of prenatal care to improve maternal and infant health.

III. INDICATED BENEFITS OF MICRONUTRIENTS ON MATERNAL MORTALITY, BIRTH WEIGHT, AND INFANT MORTALITY

Maternal mortality, birth weight, and infant mortality are complex phenomena. Maternal mortality and birth weight are predominantly influenced by the mother's nutritional status and the uterine environment. Optimal pregnancy and lactation require adjustments in maternal body composition, metabolism, physiological functions and utilization of nutrients. Meeting maternal dietary requirements is essential for the well-being of the child and for safeguarding the mother's health. The nutritional status of the fetus depends on that of the mother and this dependency may extend into childhood, particularly when the infant is exclusively breast-fed. In the case of some nutrients, the fetus is spared at the expense of the mother, whereas in the case of other nutrients, the infant will suffer more serious consequences of deficiency than the mother.

Inadequate quality of the diet, especially low consumption of animal products, is likely to result in deficiencies of iron, vitamin A, zinc, and calcium. These micronutrients are needed for many essential physiological functions, particularly for the production of enzymes and hormones that are required to regulate biological processes leading to growth, activity, development and proper functioning of the immune and reproductive systems. Micronutrients are essential for humans at all ages, but the effects of inadequate intake are particularly serious during pregnancy, lactation and infancy.

A. Vitamin A

Vitamin A is essential for women during pregnancy and lactation. Vitamin A deficiency contributes to growth deficits in infants[34] and it increases vulnerability to anemia. Nightblindness as result of vitamin A deficiency during pregnancy is common in developing countries, and is often even regarded as an early sign of pregnancy. But in fact, it is a sign of severe vitamin A deficiency. A case-control study among pregnant women in Nepal showed that women who had lower plasma levels of β-carotene and retinol were more likely to have nightblindness.[35]

The NNIPS-2 supplementation trial using a low, weekly dose of vitamin A or β-carotene among 45,000 women of reproductive age in Nepal found a 40% reduction in mortality associated with pregnancy.[2] Another beneficial effect of maternal vitamin A supplementation would be better vitamin A status in early childhood, which could be protective against infections. In addition to providing a low dose of vitamin A during pregnancy, a high-dose of vitamin A (200,000 IU) can safely be given to mothers within one month after delivery. This has been policy in Indonesia since 1992, and has been found to increase breast milk retinol levels for at least 6 months and to reduce the duration of respiratory tract infections and febrile illnesses of the breast-fed infants.[36]

Vitamin A occurs in foods in two forms. Pre-formed vitamin A, or retinol, is the readily bio-available form of vitamin A found in animal products, especially in milk, butter, cheese, egg yolk, liver and some fatty fish. Pro-vitamin A, or carotenoids, is converted to vitamin A by the body (bioconversion). But before these carotenoids are available for conversion, the digestive system has to perform an elaborate process of freeing the carotenoids from the food and making them available for absorption. This process, which determines the so-called bioavailability of a nutrient, is much more difficult for dietary carotenoids than for retinol. Carotenoids are found in plant foods, especially in green leafy vegetables and in red, orange, and yellow fruits and vegetables. Carotene bioavailability is better for fruits than for leafy vegetables.[37]

Current recommendations are to limit total vitamin A intake during pregnancy, whether from foods or supplements, to 10,000 IU per day, because of the possible teratogenic effects of higher amounts. The following doses are therefore recommended for vitamin A supplements during pregnancy, 8,000 IU/day or 25,000 IU once per week. When supplements are given in the form of beta-carotene, there will not be a teratogenic effect, because the body will not convert more dietary carotenoids into vitamin A than required.

Because of the relatively high costs of animal products and the decrease of purchasing power due to the current economic crisis, the intake of vitamin A from animal products in Indonesia is declining.[11] Vitamin A intake from plant foods seems to have remained stable. In Eastern Indonesia, the intake of vitamin A from animal products has always been low, and is even further reduced due to the economic crisis, while the consumption of vitamin A from plant foods is variable, because of limited availability, particularly in the dry season (May-September).

B. Iron

Iron has several vital functions in humans; it is the central part of hemoglobin, which carries oxygen to tissues, it is a transport medium for electrons within cells, and it plays a key role in enzyme reactions in various tissues.[38] Iron deficiency is the most prevalent nutrient deficiency in the world,[39] and it is the most important cause of anemia. Iron deficiency can be due to a low iron intake and/or to increased losses, caused by factors such as menstrual bleeding and infection, such as hookworm. Pregnant women are particularly at risk of becoming anemic because their iron requirements during pregnancy will rise to five times the non-pregnant levels. Iron deficiency anemia during pregnancy is associated with adverse outcomes, including pre-term delivery, low birth weight and mortality.[40]

Iron exists in foods in two forms, as heme iron and as non-heme iron. Bioavailability of heme iron is relatively good, while that of non-heme iron is very sensitive to absorption inhibitors (such as phytate and tannin), to absorption enhancers (such as vitamin C and red meat), and to existing iron status. Heme iron exists in red meat, while vegetables are a good source of non-heme iron. However, because vegetables usually contain absorption inhibitors, the bioavailability of their iron is relatively low. Because meat is relatively costly, especially since the start of Indonesia's economic crisis, iron intake from other sources has to be high in order to meet physiologic requirements. This makes it difficult to meet iron requirements through the diet alone, especially during pregnancy and infancy.[41] Therefore, iron supplementation is recommended for pregnant women.[42,43]

C. Folate

Pregnancy substantially increases folate requirements due to the demands of the growing fetus. Unsupplemented women are at risk for premature birth or small-for-gestational age babies. Because research has clearly shown that folate deficient mothers have a much greater risk of offspring with neural tube defects, pre-conceptual folic acid supplementation is recommended. Inadequate maternal folic acid is also associated with placental infarction,[44] and with spontaneous abortion and *abortus imminens*.[45] Lactation also causes depletion of maternal folate stores. Thus, where pregnancies closely follow one another, and where extended breast-feeding is common, maternal folate stores are likely to be compromised. Dietary sources of folate include green leafy vegetables, nuts, grains and liver, but boiling of these foods reduces their folate content. For Indonesia, folate supplementation is strongly recommended for all women of reproductive age.

D. Zinc

Zinc is essential for growth and reduces the prevalence of infectious diseases such as diarrhea and respiratory infections. Some studies have shown a positive impact of prenatal zinc supplements on birth weight.[46] Moreover, as mentioned above, prenatal zinc supplementation may improve neonatal immunological development and decrease infant morbidity.[47] A study in Indonesia of postpartum women observed that 24% were zinc deficient.[48] Diets low in bioavailable iron are also likely to be low in bioavailable zinc.

The available evidence suggests that several individual micronutrients have a beneficial impact on maternal and/or child health. Supplementation with multiple micronutrients could, therefore, result in an additive or synergistic effects. Indeed, the importance of the role of multiple micronutrients on maternal health is unquestioned in the developed world where supplementation during pregnancy is well-accepted and widespread. Still, most programs in developing countries aimed at reducing maternal and early infant mortality have tended to focus more on the health delivery issues and less on possible underlying causes, such as poor micronutrient status.

Improved understanding of the relationship between micronutrient deficiencies and their consequences for maternal and infant health and mortality could ultimately lead to appropriate and effective low-cost interventions. Along with better links between families/communities and the health care system, such interventions might promote the knowledge and skills of health-care professionals for proper diagnosis and treatment of risk factors for poor micronutrient and general nutritional status of pregnant women.

IV. CHOOSING AND EVALUATING A MICRONUTRIENT SUPPLEMENT FOR PREGNANT WOMEN

There are many opportunities for reducing maternal morbidity and mortality and for improving the nutritional status of the mothers and infants through maternal micronutrient supplementation. The most important micronutrients seem to be iron, folate and vitamin A/β-carotene, while other micronutrients such as zinc also play an important role. Therefore, a multiple vitamin/mineral supplement is indicated. The question about the impact of individual micronutrients is mainly of scientific interest, and has limited relevance for a population with a generally poor quality diet. Moreover, the additional costs of adding a few more micronutrients to an existing pre-natal supplement (e.g.

iron/folate) is marginal, and would provide women with better access to a broader range of essential micronutrients.

A recent meta-analysis of studies that compared weekly to daily iron supplementation concluded that supplementation should be continued on a daily basis.[49] In the NNIPS-2 trial, the vitamin A/β-carotene supplements were provided on a weekly basis. However, because a multi-micronutrient supplement for pregnant women should contain iron/folate, which must be taken on a daily basis, a daily supplementation schedule is also proposed for the SUMMIT intervention. The proposed multi-micronutrient supplement has recently been adopted by UNICEF following the July 1999 UNICEF/WHO/UNU Workshop, "Composition of a Multi-micronutrient Supplement to be Used in Pilot Programs Among Pregnant Women in Developing Countries." This supplement is intended for use in effectiveness trials in pilot countries, and meets the recommended daily allowances for pregnancy of the United States and Canada. The composition of the SUMMIT supplement and a summary of the workshop proceedings are included in Appendix 3. Although calcium and magnesium are absent from the supplement, this reflects the practical consideration of having supplements sufficiently small to be easily swallowed rather than any perceived lack of importance of these nutrients. Future interventions may also evaluate the impact of calcium and magnesium on pregnancy-related morbidity and mortality.

Current practice in Indonesia is for pregnant women to receive at least 90 tablets containing 60 mg iron and 250 μg folate to be taken on a daily basis as part of routine antenatal care. For the SUMMIT activity distribution of tablets will be more frequent with participants receiving tablets daily from the first antenatal clinic attendance to 90 days post-partum. The supplements will be either the multi-micronutrient combination or a matching tablet containing 30 mg iron and 400 μg folate. This strategy and formulation are proposed to maximize health benefit and compliance, and minimize any side effects. Thus, the intervention will compare the impact of a multi-micronutrient supplement for pregnant women to that of an iron/folate supplement.

V. SUPPLEMENT DISTRIBUTION

We propose to utilize the existing antenatal-care and maternal health information system as the mechanism for distributing the supplements and monitoring simple coverage rates. As mentioned above, supplements will provided daily from the first prenatal consultation for a confirmed pregnancy through 90 days post-partum. The HKI intervention staff will provide additional detailed monitoring of compliance, and also work with the midwives to increase their knowledge of the importance of maternal nutrition and strengthen their capacity for distribution of supplements, record-keeping and monitoring. To avoid over-consumption of supplements, it will be carefully emphasized to midwives and pregnant woman participating in SUMMIT that one tablet is to be taken daily, and that the supplement is in place of, and not in addition to, the normal MOH iron/folate supplement.

Use of the antenatal-care system will not only provide an opportunity for improvement of services in the area where the trial is being conducted, but will also provide valuable lessons learned for national policy and programming. Similarly, improving the existing health information system for impact monitoring will also strengthen the system. Importantly, independent monitoring of the system will be carried out by HKI to assess the reliability of the data obtained and for improving performance where necessary.

Importantly, the SUMMIT activity will not only provide information concerning the impact of micronutrients, but it will also assess the specific impact of activities accompanying the intervention such as promotion of prenatal care and social marketing. Evaluation of the relative individual effects of these factors as opposed to the multi-nutrient supplement itself will provide the basis for a cost-benefit analysis that will help the GOI to make informed decisions regarding health budget allocations. This evaluation will be achieved by first establishing a demographic surveillance system throughout the island so that district-specific maternal and infant mortality rates can be estimated. This will be followed by phased in prenatal care promotion and social marketing, and implementation of multiple micronutrient distribution.

VI. OBJECTIVES AND GOALS

The proposed SUMMIT activity focuses on decreasing maternal and early infant mortality, and increasing birth weight by improving micronutrient status during pregnancy and lactation. This will include activities aimed at increasing demand among mothers for the supplement early in pregnancy through education and social marketing, and careful monitoring of compliance. The activity will, therefore, help to improve the micronutrient status of women during and after pregnancy.

A. General objectives/goals

The primary goals of the SUMMIT activity are to determine if daily multi-micronutrient supplementation among pregnant women will:

- Reduce the frequency of maternal mortality
- Reduce the frequency of low birth weight
- Reduce the infant mortality rate

B. Specific objectives

The specific objectives will be to determine the relative impact of multi-micronutrient and iron/folate supplements on maternal and infant health, by assessing:

i. Maternal mortality defined as pregnancy-related deaths per 100,000 live births (MMR), and also pregnancy-related deaths per 100,000 pregnancies within 6 weeks of termination of pregnancy (MMRT)
ii. Birth weight
iii. Neonatal and infant mortality
iv. Maternal morbidity (e.g. hemorrhage, prolonged labor)
v. Maternal micronutrient status

VII. PROJECT ACCOMPLISHMENTS TOWARD USAID/I MISSION'S STRATEGIC OBJECTIVES

The Maternal SUMMIT activity is directly in line with USAID/I Mission's Strategic Objective, "Protecting the Health of the Most Vulnerable Women and Children" during the crisis and recovery period. The activity fully supports USAID/I's "Essential Services Component Two: Maternal and Neonatal Health (MNH)," by working through the existing health care system, to assist in "maintaining and ultimately improving the quality

and delivery of services that have proven effective in addressing the most important causes of maternal and neonatal morbidity and mortality.” The activity contributes directly to the Essential Package of Safe Motherhood Services, which focuses on “improving the clinical skills of midwives for antenatal care; birthing practices for safe delivery; appropriate management of complications, including timely referrals; identification of risk factors for neonatal mortality; care of normal and sick neonate infants; and postpartum care”. It also contributes to strengthening “demand generation and community empowerment efforts to improve birth preparedness,” strengthening “the technical and management capacity” of midwives, and supporting efforts to “improve the utilization of safe motherhood and child health services at the community level.” The activity also works toward “a decrease in the prevalence of anemia among pregnant women in project areas” (IR1: Essential Services Preserved) and an increase in the “percentage of adults knowledgeable about maternal complications of pregnancy and childbirth” (IR3: Promotion of Appropriate Behaviors and Services).

Relationship of HKI with other Organizations and the Government of Indonesia

HKI will collaborate with various departments of the Indonesian MOH at the national, provincial and district levels. HKI will continue to work closely with other USAID grantees, cooperating agencies, and contractors of USAID-funded activities in the field of maternal health and antenatal care to reduce micronutrient deficiencies, particularly iron deficiency anemia and vitamin A deficiency. The Turner Foundation (Cable News Network [CNN]) has already provided, through UNICEF, an initial US$840,000 for the Maternal SUMMIT activity to support the intervention from May 1999 until December 2000.

A Steering Committee and several Advisory Committees have been proposed at the national and local level to facilitate the work (Appendix 4). These are composed of counterparts and donor agency representatives, and includes the following organizations: MOH/Binkesmas/Gizi, MOH/NIHRD, BAPPEDA, DINKES, University of Mataram, USAID, UNICEF, WHO, and HKI.

VIII. IMPLEMENTATION

A. Schedule

The five-year implementation schedule for the trial is broadly described below:

Phase I Preparatory phase December 1999-August 2000

In this phase, we will focus on preparations for the trial, including meetings with the appropriate provincial and district level MOH staff, community leaders and midwives. A field office will be established in Mataram, the capital of Lombok. Staff will be hired and trained. Survey protocols, field manuals and data collection forms will be designed and tested, and management systems will be put in place.

At the same time efforts to increase community links to the health care system will be conducted by development of appropriate social marketing

campaigns and educational materials to increase awareness within the community and demand for the supplements.

Phase II Supplementation trial September 2000-September 2003

Implementation of the double blind, community-based, cluster-randomized, controlled trial will start. Pregnant women will be enrolled for follow up over the course of pregnancy and until three months postpartum. Once the target number of pregnancies has been enrolled, enrollment will be discontinued. Women who have not yet delivered their babies will be followed until three months after delivery.

The design, sample size, and methods are detailed below (in Section B. Implementation methods).

Phase III Analysis and Report Preparation October 2003-October 2004

The study will produce a great deal of data, requiring extensive analysis. Scientific papers, reports and presentations of the findings will be made.

B. Implementation methods

1. Study area and population

The proposed field site is Lombok Island in Nusa Tenggara Province. This site was chosen through a consensus process that evaluated qualitative and quantitative data from districts throughout Indonesia based on demographic and health indicators including maternal and infant mortality rates, nutritional status, total population, population density, health infrastructure, physical infrastructure, and local government support and stability.

Despite many years of program interventions by the GOI and NGOs, NTB remains one of the poorest provinces in the country. The UNDP development index ranked the province of NTB as 26th of 27 provinces, above only East Timor (1993 data).[50] The 1997 IDHS found that in NTB 85% of women deliver their babies at home, with 78% attended by a traditional birth attendant. Although a provincial level maternal mortality ratio (MMR) for Lombok has not yet been established, when compared to the overall rates for Indonesia, infant and under-five mortality rates for NTB are high (see Table 1).

The island of Lombok is home to approximately 2.6 million people, approximately two-thirds of the population of NTB province. The total area of the island is 4,739 km^2, and it is one of the most densely populated areas in Indonesia, with an average of 568 persons per km^2 (population densities by district: West Lombok 416/ km^2; Central Lombok 515/km^2; East Lombok 590/km^2; Kodya Mataram 5,724/km^2). The climate of the island is tropical and wet, with a relatively dry season from May to October and a wet season from November to April. Between these two main seasons are two short transitional periods. Geographically, three zones can be identified, running across the island from the northwest to the southeast: the drier and mountainous North and northwest, which is taken up in large part by the dormant volcano Gunung Rinjani; the Central zone which includes the more wet, fertile and densely populated hills and plains to the south and east of Gunung Rinjani; and the mostly dry and hilly South and Southeast.

The largest city on Lombok is Mataram, the capital of NTB province, with a population of approximately 300,000. West Lombok district consists of 13 sub districts and 99 villages with a population of around 530,000 people. Central Lombok consists of 12 sub districts and 119 villages and has a population of about 750,000 people. East Lombok consists of 16 sub districts and 109 villages with a population of about 1,000,000 people. The trial will be conducted in approximately 300 villages in West, Central and East Lombok Districts. Based on demographic data from the government of NTB and additional data collected from NGOs[51], we estimate that 72,000 pregnancies will occur annually in the 300 villages.

2. Design and Methods

The proposed SUMMIT activity is a double blind, community-based, cluster-randomized, controlled intervention designed to assess the relative impact of multi-micronutrient supplements compared to iron/folate supplements on maternal mortality, infant mortality and low birth weight. Pregnant women will randomized to one of approximately 300 clusters defined by the geographic area serviced by a trained midwife. This means that all women attended by a particular midwife will receive the same type of supplement. Clustered randomization was chosen due to the logistical and scientific difficulties associated with individual randomization in large scale longitudinal trials. Pregnant woman will be provided with either a daily multi-micronutrient supplement containing iron, folate, vitamin A, and zinc, along with additional B vitamins, minerals, anti-oxidants (for precise composition, see Appendix 3a), or an identical-looking daily supplement containing 30 mg iron and 400 μg folate. There will be no blank placebo group because iron/folate supplementation distribution to pregnant women is the current policy of the GOI.

The intervention will be preceded by a 6-month baseline demographic surveillance period to estimate area-specific maternal and infant mortality rates. Supplementation will then be phased in step-wise by implementation in 1/3 of the villages over 3 successive 3-month intervals. Women who refuse to be included in the trial and those in the pre-intervention areas will receive the standard GOI iron/folate tablets. The GOI iron/folate tablets will, therefore, not be withdrawn from the island but will only be used for individuals not enrolled in the intervention portion of the activity.

Supplements will be provided free to all women with a confirmed pregnancy, from the first antenatal consultation up to three months postpartum. As a joint activity between HKI and the GOI, the existing government system will distribute the supplements to pregnant women and the government-trained midwives will be involved in monitoring the health impact. HKI project staff will also encourage the midwives to provide maximum supplementation coverage. Additional non-invasive surveillance will be carried out by HKI project staff to allow adequate assessment of compliance and health impact.

3. Sample Size

The sample size is influenced by the minimal desirable detectable effect of the intervention, the event rate of the outcome, and the duration of the intervention. The desired detectable effect is influenced by the magnitude of the plausible effect size and the importance of the preventable event. Initial estimates of a plausible effect size of multi-micronutrient supplementation on maternal mortality can be guided by childhood

mortality-related effects observed for other micronutrients such as vitamin A and iodine, which range from 20-70%. While acknowledging that physiological differences between children and pregnant women limit the utility of such information, additional perspective is gained from the results of the NNIPS-2 study which described a vitamin A-mediated 40% reduction in mortality related to pregnancy. Given the relatively low-cost of a prenatal nutrient-based intervention, the documentation of a 20-30% effect, especially under programmatic settings, would be important. The estimates of the maternal mortality ratio in various districts on Lombok range from 400 to 800 maternal deaths per 100,000 live births. For this intervention the unit of randomization will not be the individual woman, but the area or cluster of communities administered by the midwife. This improves logistical feasibility but reduces statistical power due to the potentially correlated outcomes within each cluster. Given the estimated between-cluster variability in MMR, we have calculated that a 20% increase in sample size is needed to compensate for the loss of statistical power from the cluster-based randomization.[52] We further anticipate a loss to follow-up of approximately 10%. Lastly, because not all pregnancies result in a live birth, it is estimated that the required sample size be increased an additional 15% to compensate for abortions, miscarriage, and stillbirths. Given these factors, the sample size table below presents the number of pregnancies required to obtain significance for various observed reductions in MMR for the proposed 2-arm design.

**Detectable reduction (%)**	**Total pregnancies needed by baseline MMR (deaths/100,000 live births)**		
	**MMR=400**	**MMR=600**	**MMR=800**
15	509,635	339,137	253,887
20	281,023	187,017	140,012
25	176,230	117,290	87,815
30	119,865	79,777	59,731
35	86,232	57,381	42,967
40	64,581	43,035	32,192

Assuming a conservative MMR estimate of 400 per 100,000 live births, enrollment of 120,000 pregnancies will allow determination of a reduction in MMR of at least 30% with a power of 80% and significance level of 0.05. Similar reductions in MMRT will also be detectable because such rates are expected to be higher given that deaths prior to a live-birth delivery would be included. Given the estimated 72,000 pregnancies per year in the 300 villages on Lombok, and taking into account the phased in implementation schedule, the SUMMIT activity will plan to enroll 126,000 pregnancies over a 2 year active recruitment period. If MMR and MMRT approache 600-800 per 100,000 live births or pregnancies, which is possible considering the high rates of infant and under-five mortality, the sample size of 126,000 pregnancies will allow determination of a statistically significant reduction in MMR and also MMRT of 22-25%.

For infant mortality it will be important to also determine the age-specific effects on neonatal mortality rate (NMR, birth to 30 day mortality) and post-neonatal mortality rate

(PNMR, 1 to 12 months). The projected sample size and trial duration will allow detection of at least a 10% change in NMR and PNMR. In addition, the study will easily detect a difference of 10% in the proportion of low-birth weight (LBW) infants.

Detectable reduction (%)	Total live births (LB) needed		
	NMR=46/1000 LB	PNMR=64/1000 LB	LBW=15/100 LB
10	83,012	58,613	22,862
15	36,231	25,597	10,016
20	20,006	14,142	5,552
25	12,561	8,899	3,498

As is general practice for community-based trials, a Data Safety and Monitoring Committee will be convened, and trial data will be evaluated after two years. Should documentable reductions be likely, the committee will decide whether the trial should be terminated or extended. For example, if a 20% reduction in MMR is observed in the multi-nutrient group compared to a MMR of 600/100,000 live births in the iron/folate arm, an approximate 6-month extension would be needed to demonstrate statistical significance.

Sample size determinations are needed for the evaluation of the impact of activities, such as promotion of prenatal care and nutrition, accompanying the supplement distribution. Through the baseline demographic surveillance and continued monitoring of maternal and infant mortality as the intervention is phased in, we propose to document the effect of the activities associated with program implementation on maternal and infant mortality. This will be estimated by comparing MMR, MMRT, and IMR between the non-intervention areas and the iron/folate arm of the intervention area. For the non-intervention area we estimate that maternal and infant mortality rates will be estimated for 54,000 pregnancies. This will be compared to the rate obtained for the iron/folate arm of the intervention for 63,000 pregnancies. This will enable detection of at least a 20-25% (power=80% with $p<0.05$) effect of the program implementation, independent of the multi-micronutrient impact.

4. Demographic Baseline Survey

Prior to implementing the trial, it will be important to more fully characterize the health indicators on Lombok (i.e. MMR, MMRT and IMR) and to establish the geographic heterogeneity of such indicators. As mentioned, there are 3 distinct climatic zones with substantial heterogeneity in agricultural patterns and health seeking behavior. Additional peculiarities, such as the migration of pregnant women to their parents' house for delivery and postpartum recovery, will also be characterized. For these reasons, and to most efficiently coordinate logistics during the trial implementation phase, we will first establish a baseline demographic surveillance system for birth, death, and migration of all pregnant women and women of childbearing age (i.e. 15-45 years, as defined by the National Family Planning Board, Indonesia). This will subsequently be expanded to include children born to these women, and will eventually include other household

children and adults. This phased-in procedure prioritizes pregnant women and their children and allows estimation of several important characteristics of the trial population, including:

1. Maternal mortality ratio (MMR), and maternal mortality rate (MMRT)
2. Inter-village migration of pregnant women
3. Household composition
4. Infant mortality rate

Demographic surveillance will begin 6 months prior to the beginning of the supplement distribution and will be updated at three-month intervals during the project period. This surveillance period prior to intervention will allow estimation of maternal mortality ratio within each of the three climactic zones to within ± 20-27% (assuming an overall MMR range of 400 to 800 per 100,000 live births estimated for approximately 10,000 births per zone), and permit determination of infant mortality rates to within ± 7% in each of the three zones. This demographic database will also be linked to the HKI Nutritional Surveillance System (NSS) database, allowing the trial to track a variety of socio-economic, community and familial issues which may be associated with maternal and infant health and mortality.

5. Enrollment of pregnancies

At the onset of the supplement distribution period, pregnant women reporting to a midwife or a midwife's assistant will be enrolled into the intervention if informed consent is given. When reported to the assistant, the woman will be referred to see the midwife as soon as possible. At the time of the first visit to the midwife, the pregnancy will be confirmed by means of a urine test, or physical examination, depending on the estimated gestational age. Based on existing midwife records, we anticipate that pregnancies will be reported between 3-5 months of gestation. Reporting may be earlier once the community knows about the urine test to confirm pregnancy and about the distribution of the supplements.

Because the trial relies exclusively on pregnant women actively seeking antenatal care from the village midwife, improved midwife-seeking behavior will be important. Before the start of the trial, the midwives will be motivated to recruit three to six women in their village to assist the midwife in her trial-related tasks. The role of these assistants is to facilitate identification and enrollment of pregnant women, distribute supplements, and report complications of delivery and maternal death to the midwife. A campaign to encourage early first visits to the midwives will also be conducted in the start-up phase of the trial. The purpose of this social marketing activity is to promote early first visits, promote the use of supplements and to promote the services of the village midwife and her assistants.

6. Randomization to treatment

As described above, the unit for randomization to treatment will be the geographic area serviced by or assigned to the trained village midwife. All pregnant women living in the village of a particular midwife will receive the same supplement. Each of the 300 midwives in the distribution areas will receive a supply of one of the two supplements. Randomization of supplements to 300 midwives will be carried out by a computer

generated randomization protocol optimized to limit the occurrence of identical codes assigned to adjacent midwives. The prognostic heterogeneity and design effect of the randomization will be assessed at baseline using data collected from the demographic surveillance.

7. Supplement distribution and community cooperation

Supplementation will begin once the midwife has confirmed the pregnancy and administered the enrollment questionnaire. The supplements are to be taken daily and will be distributed in packages containing 30 tablets, as is the current MOH practice with the iron/folate tablets. The pregnant women are instructed to come back each month to obtain their next batch of tablets. Based on the current rate of antenatal clinic attendance, we anticipate that a pregnant woman will see the midwife approximately six times and will obtain supplements. If a trial participant is late for an antenatal visit, the midwife's assistants will visit the woman at home to remind her, and provide the supplements. Supplement distribution will continue until 12 weeks postpartum or 12 weeks following a miscarriage or stillbirth. In order to maximize enrollment and to ensure widespread distribution of supplements and proper recording, midwives and their assistants will receive incentives for their work on the project. In addition, targeted social marketing and educational activities will be carried out to promote use of the supplements.

8. Data collection

In addition to the distribution of supplements, midwives will perform and record the results of routine physical examinations, attend deliveries, and detect maternal and neonatal deaths. These procedures and data are already included as part of the normal MOH prenatal visit protocol. In addition, HKI data collectors will collect data at enrollment and 36 weeks during each pregnancy and at 12 weeks postpartum. The data collected on each of the sample groups is summarized below.

**Sample Group**	**Data to be collected**	**Timing of data collection**	**Data Collector**
Total sample (126,000)	Demographic baseline survey	Prior to supplementation trial	HKI data collectors
	Enrollment & informed consent	First visit to midwife	Midwife
	Socio-economic & nutrition data	Within one week of enrollment & at 36 weeks of gestation	HKI data collectors
	ANC Exam reports	ANC visits and delivery	Midwife
	Birth assessment	Delivery or within 48 hours	Midwife or HKI data collectors
	Maternal Child health visit	Seven days after delivery	Midwife
	Maternal Child health visit	12 weeks postpartum	HKI data collectors
Biochemical subgroup (20,000)	Blood samples	2 samples from woman in the subgroup drawn at specific time points of the pre- and peri-natal period	HKI venipuncturists
Maternal and infant deaths	Verbal autopsy	Within 48 hours of death	Midwife

Midwives will be encouraged to attend births in order to assist and obtain data on labor and delivery, birth weight, and APGAR scores of infants. This will be facilitated by fostering improved cooperation between trained midwives and traditional birth attendants. Although this in itself might reduce maternal mortality, this effect will be seen in both intervention groups. In addition, it will be possible to quantify the size of the mortality-reducing effect of increased attendance rates of births by midwives by comparing mortality ratios at baseline and in the iron/folate group. However, it will facilitate evaluation of the impact of multi-micronutrients on the incidence of high risk conditions, such as hemorrhage and prolonged labor, that are associated with increased risk of mortality. A final contact with the midwife is planned for seven days following delivery. The midwives will record the results of these routine examinations. In addition, data will be recorded regarding participation of women and infants in any other program aimed at improving health. This will facilitate analysis of the impact of multi-micronutrients when given in conjunction with other interventions. Procedures and forms will be standardized for the trial, and the midwives will have training and support in careful reporting.

HKI data collectors will collect data on socio-economic status, food intake (including self-acquired supplements, traditional potions, and medications) at 2 time points during each pregnancy. In addition, they will measure the height and weight of women at each visit. A final visit of the data collectors will take place three months after delivery to record information on maternal and child health. HKI data collectors will work closely with the midwives and assistants to assure that data collection is of high quality and timely. HKI workers will also collect information on midwife activity levels and community cooperation so that such information can be taken into account as an effect modifier in the analysis.

9. Biochemical determinations

At enrollment, all women will have a finger prick blood spot taken for the purpose of determining anemia using the UNICEF anemia color chart kit. This is currently utilized by many midwives and is part of the normal government pre-natal consultation protocol. A smaller number of randomly selected women will be enrolled into a sub sample of 20,000 women in which 2-3 ml venous blood will be drawn at baseline and at one other time point during pregnancy or post-delivery, resulting in 1-1.5 ml plasma from each blood draw. The time points for the second blood draw are 1 month post enrollment, 36 weeks of gestation, 1 week post delivery, and 12 weeks post delivery. This will result in 4 groups of 5000 plasma samples each. Cost, logistics, and limited plasma volume precludes the analysis of all micronutrients in each plasma specimen, nor is this statistically necessary to determine meaningful changes in micronutrient status. Therefore, each group of 5000 blood samples will be randomly sub-divided into 5 groups of 1000 samples each, with individual groups being analyzed for one of 5 specific combinations of nutrients comprised of minerals, B vitamins, carotenoids and vitamins A and E, anti-oxidants, and specific inter-related nutrients. This general approach will optimize meaningful assessment of micronutrient indicators, and their association with the measured clinical outcomes, at multiple crucial times throughout pregnancy and postpartum. This approach will allow detection of a 30% or less change (power=80%, $p<0.05$) in prevalence rates of at least 10% of individuals with low nutrient status.

Because malaria exists in many villages and is a risk factor for maternal anemia and pregnancy complications, a blood slide will also be prepared at each time point for the

subsample. This will also allow estimation of the effects of micronutrient supplementation on maternal malaria. Data on micronutrient status will also allow determination of associations between specific micronutrients and child health outcomes such as low birth weight and neonatal mortality. Blood samples will be processed in Mataram, and shipped to Jakarta for laboratory analysis.

10. Verbal autopsies

The community will be encouraged to inform the midwife as soon as possible of any complications of pregnancy or delivery, including death. Pregnancies will be tracked until a final visit at 12 weeks postpartum. Additional deaths will be detected through the on-going demographic surveillance system. This will allow the trial to include all maternal and infant deaths beyond the first 12 weeks. Through use of the existing MOH/WHO guidelines, a verbal autopsy tool will be developed, with which the midwives will determine [1] that a death which occurred was a maternal death and if possible, [2] the cause of the death. The same tool will be used by two medical doctors to independently verify the cause of death. In case of discrepancy between the results of the verbal autopsies performed by the two physicians, data will be jointly re-assessed to reach consensus. The focus of the verbal autopsy will be to ensure that a death that occurred was due to a pregnancy-related cause and occurred during pregnancy or up to 6 weeks after delivery or termination of pregnancy. This will be a crucial part of evaluating the impact of SUMMIT on MMR and MMRT.

11. Supervision, compliance and quality control

To assure high quality data collection and recording, a variety of checks and monitoring visits will be conducted. Midwives will initially receive an update training and be familiarized with specific additional aspects of data collection for the trial. The HKI maternal morbidity workers will also provide additional guidance regarding proper data reporting methodology. Assessment of compliance will be very important in this intervention and this will be monitored by midwives at each antenatal visit by pill counting. Additional HKI supplementation monitors will carry out spot-checking of pill counts on a 20% subsample each month, so that each woman in the trial will be visited at least once during the average supplementation period of 6 months. In some cases a sample pill will obtained to allow post-trial verification of allocation/distribution procedures. Assessment of social perceptions and attitudes toward pre-natal supplements, including side-effects, will also be determined by qualitative assessment methodologies. These data will be cross-referenced to the biochemical micronutrient indices determined in the subsample of women. This combination of activities will allow two compliance indices to be determined for each woman enrolled in the trial. One index will be comprised of the actual compliance data, and the other will be an estimated consumption index based on perceptions and other factors found to be predictive of use of prenatal supplements and change in micronutrient status.

12. Data management and analysis

The first check of the forms will be performed in the field by the field supervisors. Data entry will be done using the *SPSS for Windows* program. Data will be entered in batches, and 10% of each batch will be cross-checked by data-entry supervisors. Batches for which unacceptable error rates are obtained will be re-entered and checked until acceptable

quality standards are met. The data-entry program will prohibit entry of invalid or impossible values and generate a query for follow-up. After data have been entered, additional outliers will be identified and checked against the original questionnaires. Queries regarding unclear or incomplete data will be referred to the field for appropriate follow-up. This system has been field-tested by the HKI NSS and functions well. Primary analyses will compare key outcome event rates event rates, MMR, MMRT, PNMR, and LBW in the multi-micronutrient group with the iron/folate group. However, the SUMMIT activity will not only provide information concerning the impact of micronutrients on these indicators, but it will also assess the specific impact of activities accompanying the intervention such as promotion of prenatal care and social marketing. This will be done by comparison of event rates in the pre and post-intervention areas for the iron/folate arm. This enables one to individually assess the impact of the various components of the SUMMIT activity on the key health outcomes. Relative cost-benefit analyses can therefore be carried out for the various interventions.

13. Consent procedures and ethical approval

Before enrollment, all pregnant women will be informed about the project and that participation is voluntary. They will be informed that the trial will provide either the usual prenatal nutrients or the usual nutrients plus extra micronutrients. They will be asked to confirm, orally, that they understand the information given. Women who decline to participate in the trial will be provided with labeled GOI iron/folate tablets. The women that will be enrolled in the biochemical subsample will also be informed why blood samples are needed. They will be asked to consent to the blood drawing and if they understand the purpose of the blood collection. Because blood samples will be analyzed in batches and only after pairs are obtained, it is not anticipated that rapid feedback of biochemical information to the field site will be possible. Nevertheless, biochemical data indicative of a severely poor nutritional health will be sent to the field so that specific counseling or treatment can be administered.

Ethical approval for the SUMMIT activity has been obtained from the Joint Committee on Clinical Investigation of Johns Hopkins University School of Medicine, Baltimore, USA. (see Appendix 6). Approval of the protocol will also be required from the Medical Ethical Committee of the Indonesian Ministry of Health, and the intervention will only begin after such approval is granted.

14. Steering and advising committees and cooperative structure

This SUMMIT proposal is the product of teamwork involving not only HKI staff, but also a multitude of international organizations and agencies dedicated to the goal of safe motherhood and reduced maternal mortality. This collaborative approach was taken to assure that recent knowledge and lessons learned on safe motherhood and neonatal health issues have been incorporated, as well as the concerns and priorities of the Ministry of Health of Indonesia.

Successful implementation of SUMMIT activity will also require careful guidance and ongoing evaluation from multiple organizations and individuals. For these reasons, several advisory and also scientific committees have been proposed at both the local and provincial levels (see Appendix 4-1, 4-2). A full description of the committees and their

responsibilities will be detailed in the SUMMIT Manual of Operations. However a brief overview is provided below.

The Steering Committee is comprised of scientists and public health professionals at the national and international level. They will address the broad issues of the scientific and programmatic aspects of the work at the national and international level. This will include discussion and consensus-building for solutions of key scientific and programmatic issues requiring substantial changes or interventions to the trial design or implementation. It is anticipated that the Steering Committee will typically meet every 6 months for updates and discussion following recommendations and reporting from the Scientific Advisory Board. During certain periods of need the Steering Committee may be required to meet more often.

The Scientific Advisory Board is made up of scientists, administrators, and public health professionals from both the national and provincial level. This group is responsible to adequately discuss, investigate, and provide recommendations to the Steering Committee for solutions to issues regarding the success of the SUMMIT. They are also responsible for general decisions for SUMMIT that do not fundamentally change the work plan. It is anticipated that they will meet every 3 months, or more frequently if needed. The issues for discussion will be defined by the sub-committee known as the Scientific Committee.

The Scientific Committee is comprised of national and provincial scientists from the Scientific Advisory Board. They are responsible for identifying and prioritizing the key scientific issues requiring attention for the success of the SUMMIT. It is anticipated that this group will meet every 2 months for general reporting, and will frequently jointly meet with the Provincial Advisory Board and Provincial Scientific Committee.

The Provincial Advisory Board and Provincial Scientific Committee are similar in function to the Scientific Advisory Board and Scientific Committee, but for issues which are Province specific and do not require attention at the national level. These committees are primarily responsible to assure careful and coordinated implementation of the work, and identifying problem areas and making recommendations to the Scientific Committee. They will be required to meet on a monthly basis. The Provincial Scientific Committee is the group considered to be most familiar with the on-site organization and implementation of the work. They will be required to meet at least monthly and more frequently if needed.

Due to the scope of the work and its detailed nature, the implementation procedures require careful integration with existing health structures. The schematic diagram for the work (Appendix 4-1) indicates that the SUMMIT is proposed to be integrated with multiple government offices including Litbangkes within the Ministry of Health, and the provincial health and government organizations of Nusa Tenggara Barat Province including KANWIL, BAPPEDA, DINKES, and the University of Mataram, Lombok. The detailed working relationships of these groups for the SUMMIT activity will be outlined in separate Memoranda of Understanding. While it is acknowledged that HKI will be primarily responsible for the conduct of the work, it is anticipated that contributions from Litbangkes, as well as other government agencies, will be actively made in the areas of protocol and questionnaire design, certain decisions concerning staff and training, data collection activities, and supervision. The data handling and data management activities will be the primary responsibility of HKI, with analysis to be carried out with input from multiple parties. In addition, multiple agencies will be

involved with the dissemination of the central findings of the SUMMIT in both report and publication formats, and will contribute to the development of health programs arising from results of this work.

IX. RESULTS

A. Inputs

- Existing health infrastructure
- Traditional Birth Attendants
- Village Midwives
- Health Centers
- Mobilization of midwives and the community for early enrollment of pregnant mothers
- Training and mobilization of midwives for antenatal care and supplementation
- Development of materials for awareness-raising and appreciation of the supplement
- Strengthening of the maternal health information and reporting system

B. Anticipated Benchmarks

- Enrollment of 126,000 pregnant women into the antenatal care system, and provision of supplements
- Increased appreciation for maternal supplements and increased compliance
- Close collaboration with midwives to implement supplementation and reporting activities

C. Outputs

- Annual reports
- Scientific publications, presentations and seminars

D. Results

- Reduction of maternal mortality ratio and maternal mortality rate
- Regular high-quality data on maternal health
- Reduction in infant mortality
- Reduction in proportion of low birth-weight neonates

E. Proposed indicators

- Maternal mortality ratio and maternal mortality rate
- Low birth weight
- Nutritional status (height, weight and mid-upper arm circumference) of pregnant and lactating women
- Nightblindness prevalence among pregnant and lactating women
- Hemoglobin concentration among pregnant and lactating women
- Serum retinol concentration among pregnant and lactating women
- Infant mortality

X. REFERENCES

[1] From UNICEF's *State of the World's Children 1997*, found in Proceedings of the Safe Motherhood Asia 1997 Workshop, CIDA & UNICEF-sponsored event held in Ujung Pandang, South Sulawesi, Indonesia. April 6-11, 1997.

[2] West Jr KP, Katz J, Khatry SK, LeClerq SC, Pradhan EK, Shresta SR, Conner PB, Dali SM, Christian P, Pokhrel RP and Sommer A. *Double blin , cluster randomized trial of low dose supplementation with vitamin A or B carotene on mortality related to pregnancy in Nepal*. British Medical Journal 1999; 318: 570-5.

[3] Ramakrishnan U and Huffman S. *Nutrition and Health in Developing Countries*. In Semba RD, Bloem MW (eds) Nutrition and Health in Developing Countries. Humana Press, Totowa, New Jersey. Humana Press Inc. 2000.

[4] Huffman S, Baker J, Shumann J and Zehner ER. *The Case for Promoting Multiple Vitamin/Mineral Supplements for Women of Reproductive Age in Developing Countries*. Prepared for the LINKAGES Project. Washington, DC: Academy for Educational Development, November 1998.

[5] Fawzi WW, Forman MR, Levy A, Graubard BI, Naggan L, and Berendes HW. *Maternal anthropometry and infant feeding practices in Israel in relation to growth in infancy: the North African Infant Feeding Study*. Am J Clin Nutr 1997; 65: 1731-7.

[6] MOH Directorate General of Community Health, Directorate of Family Health. *Strategies to Accelerate the Reduction of Maternal Mortality*. Jakarta, 1997.

[7] From UNICEF's *State of the World's Children 1997*, found in Proceedings of the Safe Motherhood Asia 1997 Workshop, CIDA & UNICEF-sponsored event held in Ujung Pandang, South Sulawesi, Indonesia. April 6-11, 1997.

[8] Family Care International and the Safe Motherhood Inter-agency Group. *Maternal mortality, Safe motherhood fact sheet*. New York, USA, 1998.

[9] *Mother-baby Package: Implementing Safe Motherhood in Countries*. Geneva, Switzerland, World Health Organization, 1994, WHO/RHT/MSM/94.11. Rev 1

[10] Summary of maternal mortality rates in Indonesia, found in *Maternal and Neonatal Health Review with Recommendations to USAID/Indonesia*; JHPIEGO Corporation, Washington, DC; May, 1999.

[11] de Pee S, Bloem MW, Graciano F, Sari M, Soekarjo D, Tjiong R, Satoto. *Indonesia's crisis causes considerable weight-loss among mothers and adolescents.* In press.

[12] Suharno D, West CE, Karyadi D, Hautvast GAJ. *Supplementation with vitamin A and iron for nutritional anemia in pregnant women in West Java, Indonesia.* Lancet 1993;342:1325-8.

[13] Angeles-Agdeppa I, Schultink W, Sastroamidjoyo S, Gross R, Karyadi D. *Weekly micronutrient supplementation to build iron stores in female Indonesian adolescents*. Am J Clin Nutr 1997;66:177-83.

[14] Soekarjo DD, de Pee S, Bloem MW, Tjiong R, Yip R, Schreurs WHP, Muhilal. *Socio-economic status and puberty are the main factors determining anemia in adolescent girls and boys in East Java, Indonesia*. (Submitted for publication).

[15] Tanumihardjo SA, Permaesih D, Muherdiyantiningsih, et al. *Vitamin A status of Indonesian children infected with Ascaris lumbricoides after dosing with vitamin A supplements and albendazole.* J Nutr 1996;126:451-7.

[16] Central Bureau of Statistics, State Ministry of Population National Family Planning Coordinating Board, Ministry of Health and Demographic and Health Surveys Macro International Inc. *Demographic and Health Survey, 1997*, Indonesia, 1998.

[17] Cholil A, Iskandar MB, Sciortino R. *The Life Saver: The Mother Friendly Movement in Indonesia*. The State Ministry for the Role of Women, Republic of Indonesia, and the Ford Foundation, Jakarta, 1998.

[18] Summary of maternal mortality rates in Indonesia, found in *Maternal and Neonatal Health Review with Recommendations to USAID/Indonesia*; JHPIEGO Corporation, Washington, DC; May, 1999

[19] Ross JS, Thomas EL. *Iron deficiency anemia and maternal mortality*. PROFILES 3 Working Notes Series No.3 Academy of Education Development, Washington, DC; 1996.

[20] Chi, I-Cheng, Agustina T, Harbin J. *Maternal mortality at twelve teaching hospitals in Indonesia: an epidemiologic analysis*. International Journal of Obstetrics and Gynecology 1981;19:259-66.

[21] Fortney JA et al. *Maternal Mortality in Indonesia and Egypt.* World Health Organization, Geneva, Switzerland; 1985.

[22] Worthington-Roberts B. *Maternal iron deficiency and pregnancy outcome.* In Enwonwu, CO, ed., Functional significance of iron deficiency. Meharry Medical College, Nashville, TN, USA, 1990.

[23] Scholl TO, Hediger ML, Fischer RL, Schaerer JW. *Anemia versus iron deficiency: increased risk of preterm delivery in a prospective study*. Am J Clin Nutr 1992;55: 985-92.

[24] Hemminki E, and Rimpella U. *Iron supplementation, maternal packed cell volume and fetal growth*. Archives on diseases in childhood 1991;66:422-5.

[25] Roberts JM, Redman CWG. *Pre-eclampsia: more than pregnancy-induced hypertension.* Lancet 1993;341:1447-54.

[26] Crowther CA, Hiller JE, Pridmore B, Bryce R, Duggan P, Hague WM, Robinson JS. *Calcium supplementation in nulliparous women for the prevention of pregnancy-induced hypertension, preeclampsia and preterm birth: an Australian randomized trial*. FRACOG and the ACT Study Group, Aust N Z J Obstet Gynaecol 39:1, 12-8, Feb 1999.

[27] Mikhail MS, Anyaegbunam A, Garinkel D, Palan PR, Basu J, Romney SL. *Pre-eclampsia and antioxidant nutrients: Decreased plasma levels of reduced ascorbic acid, a-tocopherol, and ß-carotene in women with pre-eclampsia.* Am J Obstet Gynecol 1994;171:150-7.

[28] Ziari SA, Mireles VL, Cantu CG, Cervantes M, Idrisa A, Bobsom K, Tsin ATC, Glew RH. *Serum vitamin A, vitamin E, and ß-carotene levels in pre-eclamptic women in Northern Nigeria.* Am J Perinatol 1996;13:287-91.

[29] Merchant KM. *The impact of poor nutrition status on maternal mortality.* Final report to Safe Motherhood initiative of WHO, Geneva, Switzerland, 1991.

[30] *The effects of improved nutrition in early childhood: The Institute of Nutrition of Central America and Panama (INCAP) Follow-up Study.* Journal of Nutrition 1995;125:1027S-38S.

[31] Ceesay SM, Prentice AM, Cole TJ, Foord F, Weaver LT, Poskitt EM, Whitehead RG. *Effects on birth weight and perinatal mortality of maternal dietary supplements in rural Gambia: 5 year randomised controlled trial*. BMJ 1997;315(7111):786-90.

[32] Shankar, AH and Prasad AA. *Zinc and immune function: the biological basis of altered resistance to infection*. Am J Clin Nutr 1998;68:447S-63S.

[33] Shankar, AH, Gbakima A, Caulfield L, Zavaleta N. *The influence of maternal zinc supplementation on immunological development of the neonate and perinatal morbidity*. FASEB J. 1998;12:A4741.

[34] Underwood BA, Arthur P. *The contribution of vitamin A to public health*. FASEB J 1996 Jul;10(9):1040-48.

[35] Christian P, West Jr KP, Khatry SK, Katz J, Ram Shrestha S, Pradhan EK, LeClerq SC, Pokhrel RP. *Night blindness of pregnancy in rural Nepal - nutritional and health risks*. Int J Epidem 1998; 27: 231-7.

[36] Roy SK, Islam A, Molla A, Akramuzzaman SM, Jahan F, Fuchs G. *Impact of a single megadose of vitamin A at delivery on breastmilk of mothers and morbidity of their infants.* Eur J Clin Nutr 1997 May; 51(5):302-7.

[37] de Pee S, West CE, Permaesih D, Martuti S, Muhilal, Hautvast JGAJ. *Orange fruit is more effective than are dark-green, leafy vegetables in increasing serum concentrations of retinol and ß-carotene in schoolchildren in Indonesia.* Am J Clin Nutr 1998:68:1058-67.

[38] Hallberg L, Hulten L, Gramatkovski E. *Iron absorption from the whole diet in men: how effective is the regulation of iron absorption?* Am J Clin Nutr 1997:66;347-56.

[39] Hallberg L, Asp N (eds). *Iron Nutrition in Health and Disease.* John Libby and Company, Ltd., London, 1996.

[40] Allen LH. *Pregnancy and iron deficiency: unresolved issues.* Nutr Rev 1997 Apr;55(4):91-101.

[41] Yip R. *Iron supplementation during pregnancy: is it effective?* Am J Clin Nutr 1996; 63:853-5.

[42] Stoltzfus RJ, Dreyfuss ML. *Guidelines for the use of iron supplements to prevent and treat iron deficiency anemia.* A draft document prepared for the International Nutritional Anemia Consultative Group (INACG), 1997.

[43] World Health Organization/United Nations Children’s Fund/United Nations University. *Indicators for assessing iron deficiency and strategies for its prevention* (draft based on a WHO/UNICEF/UNU Consultation, 6-10 December 1993). WHO, 1993.

[44] Hall J, Solehdin F. *Folic acid for the prevention of congenital anomalies*. Eur J Pediatr 1998 June; 157(6):445-50.

[45] Pietrzik KF, Thorand B. *Folate economy in pregnancy*. Nutrition 1997;13(11-12):975-7.

[46] Ramakrishnan, U., R. Manjrekar, J. Rivera, T. Gonzales-Cossio and R. Martorell. *Micronutrients and pregnancy outcomes: A review of the literature*. Nutrition Research 1999; 19:103-139.

[47] Caulfield, LE, Zavaleta N, Shankar AN, Merialdi M. *The potential contribution of maternal zinc supplementation during pregnancy to maternal and child survival.* Am J Clin Nutr 1998; 68:499S-508S.

[48] Wieringa FT, Dijkhuizen MA and Van der Meer J. *Vitamin A, zinc and iron deficiency in mothers and infants in Indonesia.* IVACG Abstracts. Cairo, 1997.

[49] Galloway R, McGuire. *Daily versus weekly: how many iron pills do pregnant women need?* J Nutr Rev 1996; 54:318-23.

[50] Directorate General of Human Settlements, Ministery of Public Works, GOI / World Bank. Lombok Integrated Urban Infrastructure Development Project. Unpublished report. 1999.

[51] NTB Provincial Office of the Department of Health, GOI / PATH. *Study Protocol for Hib vaccine trial in Lombok,* 1998.

[52] Kirkwood, BR. *Essentials of Medical Statistics*. Blackwell Scientific Publications, 1988, p196.

Appendix A

Appendix A

XIII. APPENDICES

1. Brief description of HKI
2. Research site
3. Composition of the multi-micronutrient supplement
4. SUMMIT organizational charts
5. Timeline of activities
6. Johns Hopkins University Ethical approval papers and sample consent form

Appendix A

SUMMIT Activity Committees- Appendix 4-2

STEERING COMMITTEE

CP	Dr. Sri Astuti S. Suparmanto	Head of the National Institute of Health Research and Development, Ministry of Health, Republic of Indonesia
	Prof. Dr. Azrul Anwar	Director General Public Health, Ministry of Health
	Prof. Dr. Farid A. Moeloek	OBGYN, Former Minister of Health of the Republic of Indonesia
	Dr. Martin W. Bloem	Regional Director, HKI Asia-Pacific/Country Director, HKI Indonesia
	Dr. Arum Atmawikarta, MPH	Head of Nutrition Research and Development Center, Ministry of Health, Republic of Indonesia
	Dr. Anuraj Shankar	SUMMIT Program Director, HKI Indonesia
	Dr. Dini Latief, MSc	Director of Nutrition, Ministry of Health, Republic of Indonesia
	Dr. Fasli Jalal	Head of Bureau Kesehatan Gizi Masyarakat BAPPENAS
	Prof. Dr. Darwin Karyadi	Regional Director, SEAMEO/TROPMED
	Dr. Richard Guidotti	Medical Officer, Reproductive Health and Research, WHO, Geneva
	Dr. Roger Shrimpton	Head of Nutrition, UNICEF, New York
	Ms. Molly Gingerich	Head of Public Health and Nutrition, USAID Indonesia
	Dr. Ray Yip	UNICEF/CDC

SCIENTIFIC ADVISORY BOARD

CP	Dr. Anuraj Shankar	SUMMIT Program Director HKI Indonesia
	Dr. Arum Atmawikarta, MPH	Head of Nutrition Research and Development Center, Ministry of Health, Republic of Indonesia
	Dr. Dini Latief	Director of Nutrition, Ministry of Health, Republic of Indonesia
	Dr. Muhilal	Nutrition Research and Development Center, Ministry of Health, Republic of Indonesia / Yayasan Gizi Bogor
	Dr. Martin Bloem	HKI-Asia/Pacific Regional Director, HKI Country Director-Indonesia
	Dr. Saskia de Pee	HKI Indonesia
	Prof. Dr. Soemilah Sostroamidjojo	SEAMEO/TROPMED
	Dr. Dwi Susilowati	SEAMEO/TROPMED
	Mr. Sunawang	Head of Nutrition, UNICEF Indonesia
	Dr. Abas Basuni	Nutrition Research and Development Center, Ministry of Health, Republic of Indonesia
	Prof. Dr. Mulyanto	Rector of University of Mataram
	Dr. Mas Irwan Singagerda	Head of the Provincial Office of the Ministry of Health, NTB
	Dr. Soesbandoro	Head of Mataram General Hospital, OBGYN
	Dr. Husni Mu'adz	University of Mataram, NTB
	Dr. Hananto Wiryo	Pediatrician, NTB
	Dr. Damayanti Soekarjo	SUMMIT Field Director, HKI Indonesia

Appendix A

SUMMIT Activity Committees- Appendix 4-2 (continued)

SCIENTIFIC COMMITTEE

CP	Dr. Anuraj Shankar	SUMMIT Program Director HKI Indonesia
	Dr. Arum Atmawikarta, MPH	Head of Nutrition Research and Development Center, Ministry of Health, Republic of Indonesia
	Dr. Muhilal	Nutrition Research and Development Center, Ministry of Health, Republic of Indonesia / Yayasan Gizi Bogor
	Dr. Martin Bloem	HKI-Asia/Pacific Regional Director, HKI Country Director-Indonesia
	Dr. Dwi Susilowati	SEAMEO/TROPMED
	Dr. Abas Basuni	Nutrition Research and Development Center, Ministry of Health, Republic of Indonesia
	Prof. Dr. Mulyanto	Rector of University of Mataram
	Dr. Soesbandoro	Head of Mataram General Hospital, OBGYN
	Dr. Husni Mu'adz	University of Mataram, NTB
	Dr. Damayanti Soekarjo	SUMMIT Field Director, HKI Indonesia
	Dr. Hananto Wiryo	Pediatrician, NTB

PROVINCIAL ADVISORY BOARD

Drs. Harun Al Rasyid	Governor, NTB
Prof. Dr. Mulyanto	Rector of University of Mataram
Dr. Mas Irwan Singagerda	Head of Provincial Office Ministry of Health, NTB
Dr. Aswandono	Head of Dinas Kesehatan NTB
Ir. Nanang Samodra	Head of Provincial Planning Agency, NTB
Dr. Soesbandoro	Head of Mataram General Hospital, OBGYN
Dr. Husni Mu'adz	University of Mataram, NTB
Dr. Anuraj Shankar	SUMMIT Program Director
Dr. Agus Sutanto	Head of Bidang Desentralisasi dan Tugas Bantuan, Provincial Office Ministry of Health, NTB
Dr. Damayanti Soekarjo	SUMMIT Field Director, HKI Indonesia
Dr. Hananto Wiryo	Pediatrician, NTB

PROVINCIAL SCIENTIFIC COMMITTEE

Prof. Dr. Mulyanto	Rector of University of Mataram
Dr. Husni Mu'adz	University of Mataram, NTB
Dr. Hananto Wiryo	Pediatrician, NTB
Dr. Soesbandoro	Head of Mataram General Hospital, OBGYN
Dr. Anuraj Shankar	SUMMIT Program Director
Dr. Damayanti Soekarjo	SUMMIT Field Director, HKI Indonesia
Dr. Agus Sutanto	Head of Bidang Desentralisasi dan Tugas Bantuan, Provincial Office Ministry of Health, NTB
(to be determined)	representative from Dinas Kesehatan NTB
(to be determined)	religious leader, Lombok

AD-HOC ADVISORS

Dr. Djelantik	Pediatrician, Lombok
Prof. Dr. Suwignyo	Head of Laboratory in Mataram General Hospital
(to be determined)	heads of local NGOs
(to be determined)	religious leaders
(to be determined)	adat leaders

Appendix A

SUMMIT Activity Committees-Appendix 4-2 (Continued)

HKI Maternal Mortality Technical Team

Dr. Martin Bloem	HKI-Asia/Pacific Regional Director, HKI Country Director-Indonesia
Dr. Ian Darnton-Hill	Vice President of Programs, HKI New York
Dr. Anuraj Shankar	SUMMIT Program Director HKI Indonesia
Dr. Regina Moench-Pfanner	Regional Coordinator, HKI Asia-Pacific
Ms. Lynnda Kiess	NSS Program Director, HKI-Asia/Pacific
Dr. Roy Tjiong	Deputy Director and Medical Director, HKI Indonesia
Dr. Saskia de Pee	HKI Indonesia
Ms. Mayang Sari	Head of Nutrition, HKI Indonesia
Dr. Amy Rice	Vitamin A Program Director, HKI Indonesia
Dr. Damayanti Soekarjo	SUMMIT Field Director, HKI Indonesia
