# Supplementary material for: The Effect of Maternal Multiple Micronutrient Supplementation on Cognition and Mood during Pregnancy and Postpartum in Indonesia: A Randomized Trial
Source: PLoS One. 2012 Mar 12;7(3):e32519. doi: 10.1371/journal.pone.0032519 (PMC3299672; doi:10.1371/journal.pone.0032519)
Supplement: Appendix S1 — The contents of the two maternal supplements. (DOC) [file pone.0032519.s003.doc]

Appendix S1. The contents of the two maternal supplements.

Iron and Folic Acid (IFA):

30 mg iron (ferrous fumarate)

400 μg folic acid

Multiple Micronutrients (MMN) :

30 mg iron

400 μg folic acid

800 μg retinol (retinyl acetate)

200 IU vitamin D (ergocalciferol)

10 mg vitamin E (alpha tocopherol acetate)

70 mg ascorbic acid

1.4 mg vitamin B1 (thiamine mononitrate)

1.4 mg vitamin B2 (riboflavin)

18 mg niacin (niacinanide)

1.9 mg vitamin B6 (pyridoxine)

2.6 μg vitamin B12 (cyanocobalamin)

15 mg zinc (zinc gluconate)

2 mg copper

65 μg selenium

150 μg iodine
